# NFκB1: a common biomarker linking Alzheimer's and Parkinson's disease pathology

**DOI:** 10.3389/fnins.2025.1589857

**Published:** 2025-05-06

**Authors:** Adam Cunningham, Emma Barrett, Sebastian Risch, Peter H. U. Lee, Chan Lee, Abhay Moghekar, Prabir Patra, Joon W. Shim

**Affiliations:** ^1^Department of Biomedical Engineering, Marshall University, Huntington, WV, United States; ^2^Department of Cardiothoracic Surgery, Southcoast Health, Fall River, MA, United States; ^3^Department of Pathology and Laboratory Medicine, Brown University, Providence, RI, United States; ^4^Department of Anesthesiology, Perioperative, and Pain Medicine, Brigham and Women's Hospital and Harvard Medical School, Boston, MA, United States; ^5^Department of Neurology, Johns Hopkins School of Medicine, Baltimore, MD, United States

**Keywords:** Alzheimer's disease, Parkinson's disease, mitochondria, neuroinflammation, caudate nucleus (CN)

## Abstract

**Introduction:**

Alzheimer's disease (AD) and Parkinson's disease (PD) are neurodegenerative disorders characterized by mitochondrial dysfunction and chronic inflammation. The transcription factor NF-κB1 is implicated in both neuroprotective and pro-inflammatory processes, with its activity varying between neurons and glial cells. While previous studies have explored the genetic and epigenetic contributions to these diseases, the infection hypothesis has re-emerged as a potential framework for identifying novel biomarkers and therapeutic targets.

**Methods:**

We conducted bulk RNA sequencing on human postmortem caudate nucleus tissue samples obtained from cognitively normal controls (*n* = 5), AD patients (*n* = 6), and PD patients (*n* = 3). Differential gene expression analysis and pathway enrichment were performed to identify dysregulated signaling mechanisms relevant to neuroinflammation and mitochondrial function.

**Results and discussion:**

TNFα signaling through the NF-κB pathway was identified as a prominently dysregulated mechanism in both AD and PD samples. Transcript levels of NFE2L2 (NRF2) and NF-κB1 were elevated, coinciding with reduced expression of the mitochondrial transporter gene SLC25A6, suggesting a compensatory response to oxidative stress. Additionally, PLCG2 expression was markedly increased in microglial populations, reflecting heightened immune activation. A significant 10-fold reduction in hemoglobin subunit alpha (HbA1) RNA was observed in disease groups compared to controls, indicating compromised oxygen transport and cellular stress. These findings highlight candidate biomarkers and suggest that therapeutic strategies targeting mitochondrial integrity and neuroinflammation may be effective in AD and PD.

## Introduction

Alzheimer's Disease (AD) and Parkinson's Disease (PD) are two of the most common neurodegenerative disorders, affecting millions worldwide (Chopade et al., 2023). AD impacts over 50 million people (Velandia et al., 2022), a number expected to triple by 2050 (Bloem et al., 2021; Ou et al., 2021). PD affects over 10 million globally (Bloem et al., 2021; Ou et al., 2021). Both diseases cause cognitive and motor dysfunction, largely driven by mitochondrial dysfunction (Prasuhn and Bruggemann, 2021; Sharma et al., 2021). The economic burden is immense: AD alone costs $305 billion annually in the U.S., projected to exceed $1 trillion by 2050 (2020; 2021; 2022; 2023). Similarly, PD incurs substantial medical and indirect costs, severely impacting patients (Salamon et al., 2020; Bloem et al., 2021; Merello et al., 2021; Garcia-De-La-Fuente et al., 2022), caregivers, and society, with significant psychological and social repercussions (Cross et al., 2018).

The most urgent medical need in AD and PD is to develop therapies to halt or reverse disease progression. In doing so, detecting key factors linked to brain metabolic waste is crucial. NF-κB plays a central role in both AD (Lukiw, 2022; Sun et al., 2022; Patel et al., 2024; Soelter et al., 2024) and PD (Li et al., 2012; Jafari et al., 2022), regulating inflammation, cell survival, and immune responses. In AD, NF-κB is implicated in amyloid-beta plaque formation and tau hyperphosphorylation (Sun et al., 2022), while in PD, it contributes to dopaminergic neuron loss (Rivas-Arancibia et al., 2015). NF-κB activation is linked to mitochondrial dysfunction and chronic neuroinflammation, exacerbating neurodegeneration in both diseases (Lin et al., 2022). While the phenotype of NFkB1 knockout mice is well documented (Cartwright et al., 2016), the RNA level of this transcription factor in the aged human brain with AD or PD (Lin et al., 2024) is poorly understood.

In the case of AD, the disease course for the individual patient can be varied, typically following several stages from preclinical stage to mild/moderate/severe stage through mild cognitive impairment (MCI) with about 90–95% patients diagnosed as late-onset (Zhang et al., 2020). Despite the prevalence of AD and PD, the shared pathophysiology of these two is still poorly understood. The latest findings offer evidence highlighting the potential clinical benefits of semaglutide in reducing the risk of AD onset and progression but how brain glucose level affects cognition through which mechanism is less clear (Wang et al., 2024).

The brain is highly glucose-dependent, and microorganisms can utilize glucose as an energy source. One of the key brain structures involved in regulating cognitive function through learning and memory is the caudate nucleus (CN), making it a priority target for diagnosing and treating AD and Parkinson's dementia (PDD). As these conditions are considered polygenic diseases (Arena et al., 2024; Beydoun et al., 2024; Faouzi et al., 2024; Gabbert et al., 2024; Loesch et al., 2024; Shi et al., 2024; Tunold et al., 2024; Kals et al., 2025; Leffa et al., 2025; Tan et al., 2025), pathogens can enter the brain through the olfactory pathway, blood-brain barrier (BBB) disruption, or neuroinflammation, which facilitates microbial persistence and immune activation. Further, bacterial toxins disrupt mitochondrial function (Lobet et al., 2015). These factors suggest that if bacteria reach the brain (MacDonald, 1986; Dickson, 1987; MacDonald and Miranda, 1987), the CN could be a favorable niche for their survival and maintenance.

The glucagon-like peptide-1 receptor (GLP-1R), solute carrier family 25 member 6 (SLC25A6) in mitochondria, and solute carrier family 9 member 9 (SLC9A9), which regulates endosomal pH, are crucial genes in cellular metabolism, whose dysfunctions are linked to the pathogenesis of AD and PD (Maskery et al., 2020; Pedersen and Counillon, 2019; Manfready et al., 2022). Previous studies showed reduced GLP-1R RNA in the caudate nucleus in AD (Barrett et al., 2024). A Phase II trial found GLP-1R agonist, Lixisenatide, improved motor function in early PD (Meissner et al., 2024). Evidence suggests GLP-1R agonism may benefit cognitive and motor functions in AD and PD, possibly through mitochondrial restoration (Zhao and Pu, 2019). Growing evidence suggests GLP-1R (Barrett et al., 2024), SLC25A6 (Palmieri, 2004), and SLC9A9 (Fuster and Alexander, 2014) are related to mitochondria in AD and PD.

Despite accruing evidence suggesting that GLP-1R agonists may benefit cognitive and motor functions in AD and PD (Holscher, 2022), it remains unclear which mitochondrial markers (Tomas et al., 2011; Morales et al., 2014; Holscher, 2024) can explain their efficacy in both conditions. To address this gap, we focus on the CN, a brain region implicated in both AD and PD pathology and highly involved in cognitive and motor processing. By conducting whole transcriptome RNA-Seq of the CN forming part of the basal ganglia in AD and PD, our approach aims to identify critical gene alterations that impact mitochondrial function, ER stress, and endosomal activity along with changes associated with key risk genes for AD (Scheltens et al., 2021), providing new insights into the molecular mechanisms underlying these diseases and the therapeutic effects of GLP-1R agonists.

## Results

**Figure 1:**
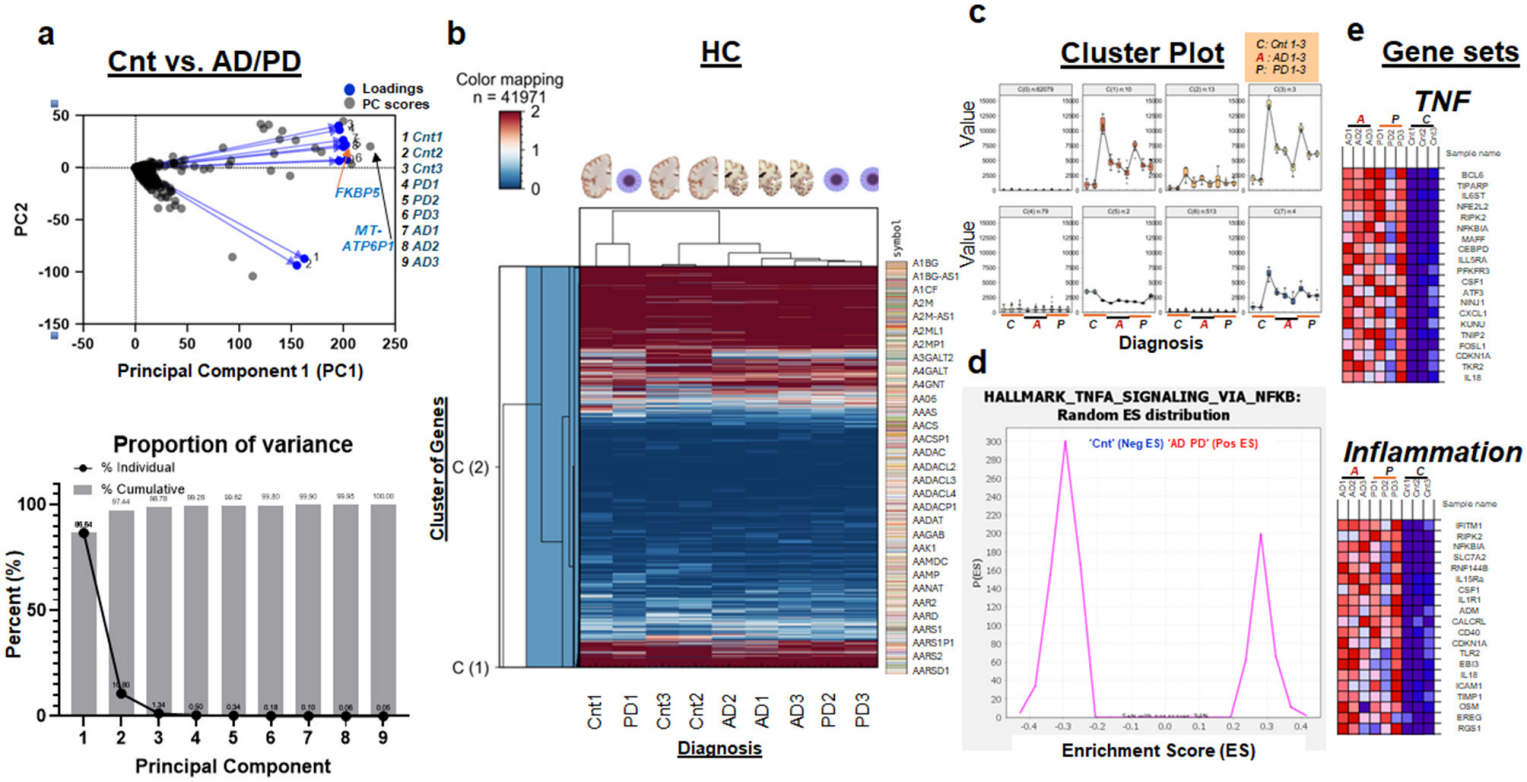
Integrated Analysis of Gene Expression Profiles in AD and/or PD (AD/PD). **(a)** Top: PCA biplot of caudate nucleus specimens from control and subjects with AD and/or PD. Regarding PC scores: A datapoint with a high positive score on PC1 (MT-ATP6P1) indicates that it aligns strongly with the pattern or trend represented by PC1. Regarding Loadings: If the original variables X1 and X2 have high positive loadings on PC1, a datapoint (i.e., FKBP5) that is far right on PC1 is likely to have high values for X1 and X2. Bottom: Proportion of variance plot showing PC1 accounting for 86.6% of variances. **(b)** Hierarchical Clustering of Gene Expression Profiles from Control and AD/PD Specimens: This figure presents a hierarchical clustering dendrogram based on the analysis of 41,971 data points from gene expression profiles of caudate nucleus specimens. The *x-*axis denotes the samples involved in the study. This analysis aids in visualizing the genetic distinctions between control and AD/PD groups, potentially highlighting key pathways involved in the disease's pathogenesis. **(c)** Cluster plot analysis of gene expression across eight subgroups in control and AD/PD specimens. Key findings between control and disease groups can be highlighted by focusing on cluster plots with varying *y-*axis values, including C(1), C(2), C(3), C(5), and C(7). **(d)** Distribution of Enrichment Scores (ES) for Hallmark TNF alpha signaling. Gene sets highly expressed in AD/PD have fewer candidates (right triangle) than those in the control group (left triangle). Gene set analysis highlights the importance of the TNF-alpha pathway via NFkB. Check TNFα, NFkB, and downstream molecules; NFkB1 is significantly different (see Figure 2g). **(e)** Comparative analysis of gene expression in TNF and inflammation pathways between control and AD/PD specimens: This figure illustrates the expression levels of two key gene sets—TNF and Inflammation—in control and AD/PD specimens.

To elucidate the complexities of gene expression in neurodegenerative disorders, we employed Principal Component Analysis (PCA) on large-scale RNA-seq data derived from human postmortem tissues (type: the CN; the patients-all white at median age 75 years; one of two batches at *n* = 3 in controls; *n* = 3 in AD; *n* = 3 in PD). This analytical technique was pivotal in reducing the dimensionality of the data while preserving the most significant variances inherent in the dataset. Our PCA results, which were carried out on data obtained from the combined RNA-seq analysis of AD and PD samples, identified FKBP and MT-ATP6P1 as the genes exhibiting the most variation along the primary component (PC1), implicating a strong mitochondrial involvement (Figure 1a). This finding underscores the potential role of mitochondrial dysfunction in the pathological landscape of AD (Supplementary Figure S1) and PD (Supplementary Figure S2) alike, for the following reasons. When the AD and PD groups of postmortem specimens were combined for PCA as AD/PD, MT-ATP6P1 exhibited the highest variation along PC1 (Figure 1). However, one control specimen (Control 3) appears transcriptomically similar to the AD/PD group in this RNA-seq batch, representing a confounding limitation of the PCA—where 33% of control samples exhibit an AD/PD-like phenotype (Figure 1a).

Separating the PCA for AD and PD revealed a common gene along PC1: CYTB (Supplementary Figures S1, S2), which encodes cytochrome b, a key component of the mitochondrial cytochrome bc1 complex (Complex III) in the electron transport chain, essential for cellular respiration and ATP production. The presence of CYTB in both datasets (Supplementary Figures S1, S2) highlight its mitochondrial significance. Further, our study utilized hierarchical clustering to dissect the patterns of gene expression between control and disease states—AD and PD. Interestingly, the gene expression profiles did not segregate strictly along diagnostic lines, revealing a nuanced interplay of 41,971 transcripts, indicative of the underlying biological complexity (Figure 1b).

Cluster plot analyses (Figure 1c) and enrichment score evaluations (Figure 1d) revealed significant alterations in TNFα signaling and inflammation (Figure 1e) pathways in AD/PD (Figures 1c–e; Supplementary Figures S1c–e, S2c–e). These findings highlight the critical role of inflammation in disease mechanisms and provide a refined understanding of the molecular dynamics in AD and PD. The insights raise an important question: which specific inflammatory markers are altered in these neurodegenerative conditions?

**Figure 2:**
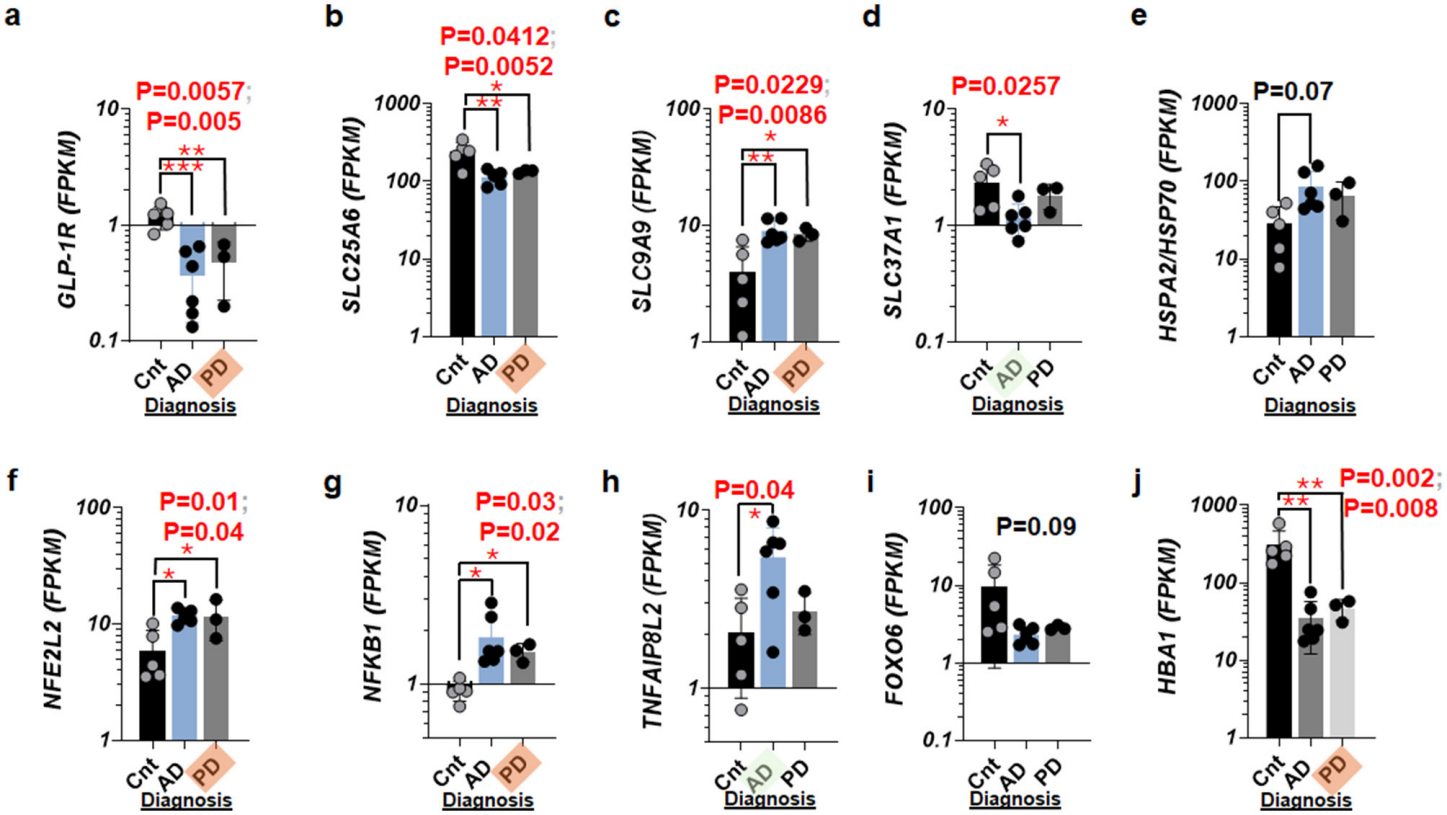
Elevated NFkB1 and concurrent impacts on GLP-1R and genes encoding SLC proteins in AD and PD. **(a)** Scatter plots with bar graphs showing GLP-1R RNA activity in the caudate nucleus with AD and PD as compared to control (Cnt) specimens. **(b)** Scatter plots with bar graphs showing SLC25A6 in the caudate nucleus with AD and PD. **(c)** Scatter plots with bar graphs showing SLC9A9 in the caudate nucleus with AD and PD. **(d)** Scatter plots with bar graphs showing SLC37A1 in the caudate nucleus with AD and PD. **(e)** Scatter plots with bar graphs displaying HSPA2 RNA activity in the caudate nucleus with AD/PD. **(f)** Scatter plots with bar graphs displaying the transcription factor NFE2L2 RNA activity in the caudate nucleus with AD and PD. **(g)** Scatter plots with bar graphs showing the transcription factor NFkB1 RNA activity in the caudate nucleus with AD and PD. **(h)** Scatter plots with bar graphs displaying TNF-related gene encoding TNFAIP8L2 RNA activity in the caudate nucleus with AD and PD. **(i)** Scatter plots with bar graphs displaying the transcription factor FOXO6 RNA activity in the caudate nucleus with AD and PD. **(j)** Scatter plots with bar graphs showing RNA activity of HBA1 (hemoglobin subunit protein gene) in the caudate nucleus with AD and PD. When both AD and PD show statistical significances, an orange marker highlights the tilted label, PD, on the *x-*axis. When AD group alone is significant, the green marker shines the tilted label, AD. *P* < 0.05 (^*^), *P* < 0.01 (^**^), and *P* < 0.005 (^***^).

To further delineate the specificity of these molecular changes, we segmented the pooled disease groups (Figure 1) into distinct AD and PD categories for a comparative analysis (Figure 2). This analysis revealed that while GLP-1R, SLC25A6, and SLC9A9 showed consistent alterations across both diseases relative to controls (Figures 2a–c), SLC37A1 exhibited more specific changes to AD (Figure 2d). Unlike the preliminary screen (Batch 1 dataset at *n* = 3 per group), HSPA2 did not achieve statistical significance when comparing individual diseases to the control group (Figure 2e). To better understand the underlying mechanisms, we checked eleven candidate transcription factors with TNF-related gene and hemoglobin gene, HBA1 (Figures 2h–j; Supplementary Figure S3) and found that NFE2L2 encoding NRF2 and NFkb1 were significantly elevated in the CN of AD and that of PD as compared to that of controls (Figures 2f, g), while TNFAIP8L2 was elevated in AD alone (Figure 2h). Strikingly, a roughly one order of magnitude reduction (10-fold, 305 vs. 35 FPKM) in HBA1 RNA activity was detected in the CN of AD specimens, while a 7-fold decrease (305 vs. 46 FPKM) was observed in HBA1 RNA of PD specimens compared to controls (Figure 2j).

Given the unusual nucleotide sizes of GLP-1R in four different species (Fig.S4) (Barrett et al., 2024), the gene set enrichment analyses (Supplementary Figure S5) suggested that in addition to GLP-1R, RNA activities of SLC25A6 and NFkB1 were significantly altered in AD and PD alike (Figures 2b–g).

**Figure 3:**
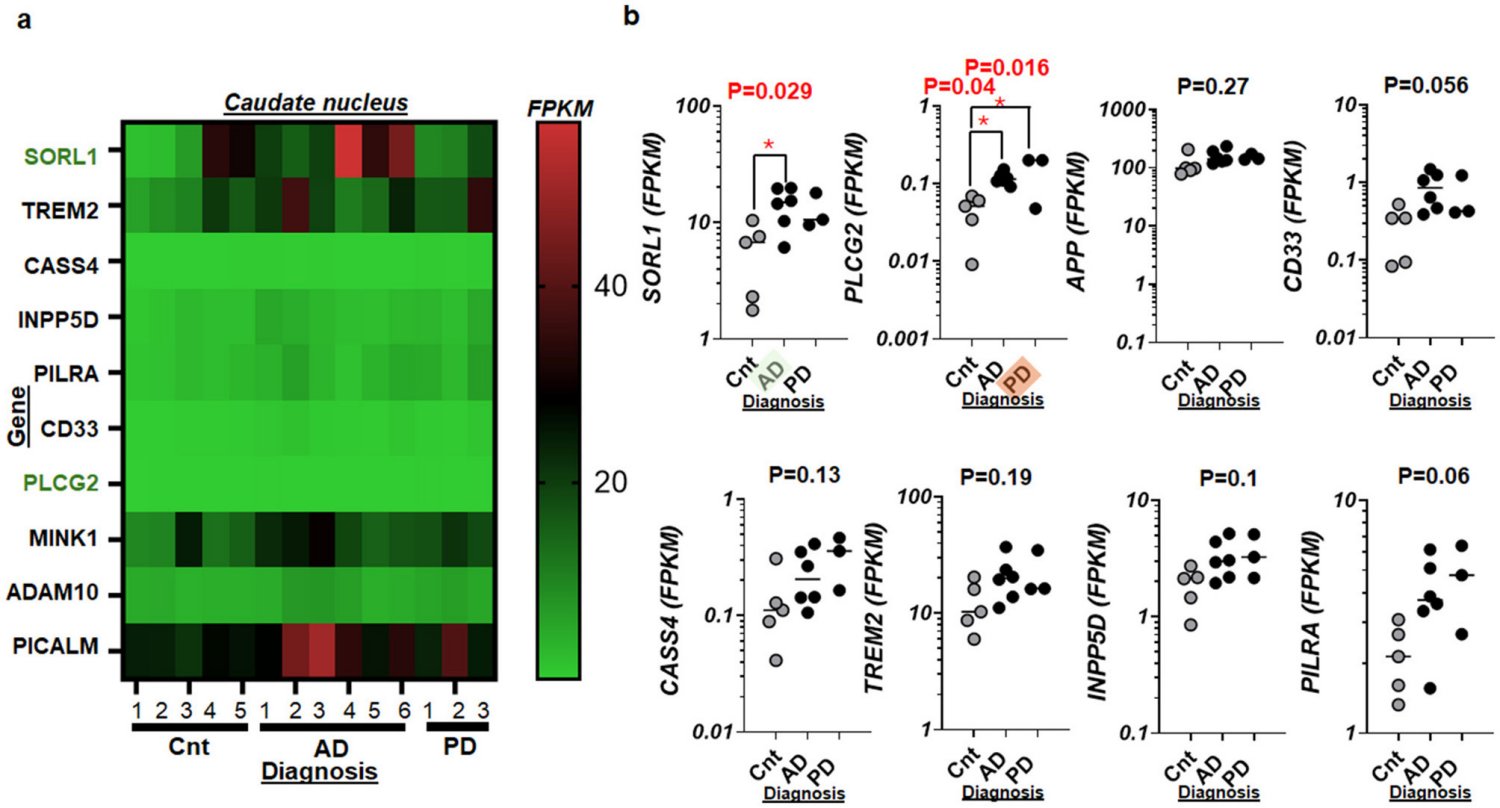
Select risk genes of AD and their RNA activities in the human postmortem brains with AD and/or PD. **(a)** Heat map displaying an overall view of whole transcriptome RNA-Seq, involving SORL1 and nine other genes suggested previously (Scheltens et al., 2021) in the caudate nucleus of control (CNT, *n* = 5), AD (*n* = 6), and PD (*n* = 3). **(b)** Scatter plots showing RNA activities of SORL1, PLCG2, APP, CD33, CASS4, TREM2, INPP5D, and PILRA in the caudate nucleus with AD and PD as compared to that of control (Cnt) specimens. When both AD and PD show statistical significances, an orange marker highlights the tilted label, PD, on the *x-*axis. When AD group alone is significant, the green marker shines the tilted label, AD. ^*^P < 0.05.

To deepen the understanding of genetic contributions to AD as well as to PD, we focused on the previous report revealing AD risk genes (Scheltens et al., 2021) for their potential roles in both disorders (Supplementary Figures S6–S10). A comprehensive analysis of these genes within the CN revealed significant alterations in their RNA activities in AD as well as in PD cases compared to controls (Figures 3a, b). Notably, the RNA level of PLCG2 was significantly elevated in the CN of AD and that of PD as compared to that of controls, while SORL1 demonstrated a significant elevation only in AD (Figure 3b). The transcript size of SORL1 displayed consistency across multiple species while the same is not true for PLCG2 or longer in humans compared to rodents (Supplementary Figure S10a), consistent with GLP-1R (Supplementary Figure S4). Other than SORL1 and PLCG2, however, we did not detect significant alterations of AD risk genes when AD and/or PD specimens were compared to those of controls (Figure 3b). The specific expression patterns of SORL1, primarily linked to memory in hippocampus and the CN in the AD brain (Tejada Moreno et al., 2022), along with elevated PLCG2 indicative of reactive microgliosis (Andreone et al., 2020) in the brain with AD as well as PD demonstrate the molecular complexity of these diseases in which previously reported AD risk genes and PD-associated markers failed to show statistical significances (Supplementary Figures S6–S11, S17).

**Figure 4:**
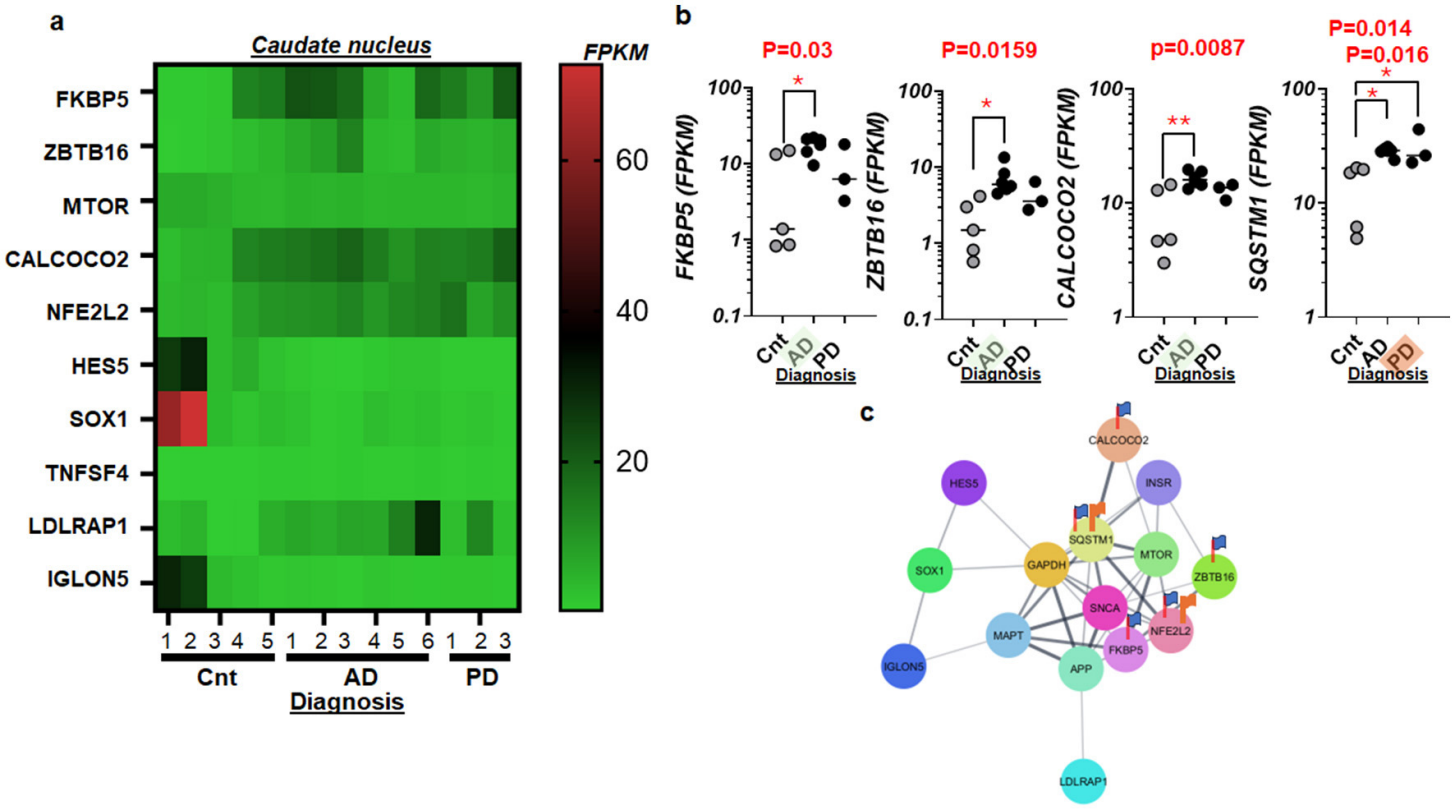
Stress response genes and their RNA activities in the human postmortem brains with AD and/or PD. **(a)** Heat map displaying an overall view of whole transcriptome RNA-Seq, involving FKBP5 and nine other genes in the caudate nucleus of control (CNT, *n* = 5), Alzheimer's disease (AD, *n* = 6), and Parkinson's disease (PD, *n* = 3). **(b)** Scatter plots displaying RNA activities of FKBP5, ZBTB16, CALCOCO2, and SQSTM1 in the caudate nucleus with AD and PD as compared to that of control (Cnt) specimens. When both AD and PD show statistical significances, an orange marker highlights the tilted label, PD, on the *x-*axis. When AD group alone is significant, the green marker shines the tilted label, AD. *P* < 0.05 (^*^) and *P* < 0.01 (^**^) **(c)** protein network of genes shown in **(a, b)**. Note that a flag represents statistical significance.

To further explore the molecular basis of stress impacts in the aged brain afflicted by AD and PD, we conducted a detailed analysis of RNA activities for FKBP5 and nine other genes (Figure 4; Supplementary Figures S11–S16). These genes were selected based on their prominent effect sizes and statistical significances, ranking them within the top 100 for their potential relevance in neurodegenerative pathways. Notably, we observed significant increases in the RNA activities of SQSTM1, associated with NF-kB signaling (Zou et al., 2020), in the CN of AD and PD patients (Figure 4b), while those of FKBP5, ZBTB16, and CALCOCO2 were elevated in AD alone (Figures 4a–c). Such elevations might indicate overwhelming responses to excessive stress in PD or AD.

Furthermore, we examined the nucleotide length consistency of FKBP5 and NFE2L2 across four different species—mice, rats, chimpanzees, and humans. The results (Supplementary Figure S11b), highlighted a remarkable conservation of nucleotide lengths among these species, suggesting a fundamental role for these genes in neural function that is preserved across evolutionary boundaries. These findings shed light on the complex genetic landscape of AD and PD, particularly regarding how genes involved in stress response mechanisms are altered (Supplementary Figures S12–S16). Understanding these changes is crucial for developing targeted interventions that could bolster the brain's resilience against the neurodegenerative impacts of chronic stress.

To investigate neurotransmitter regulation in the aging brain affected by AD and PD, we analyzed the RNA activities of key neurotransmitter receptors. These receptors included DRD1, DRD2 (dopamine receptors), GLP1R (glucagon-like peptide 1 receptor), GHSR (growth hormone secretagogue receptor), OPRM1, OPRD1, OPRK1 (opioid receptors), and HTR1A, HTR2A (serotonin receptors). Our comparisons focused on discerning how these neurotransmitters influence neurodegenerative processes within the CN.

**Figure 5:**
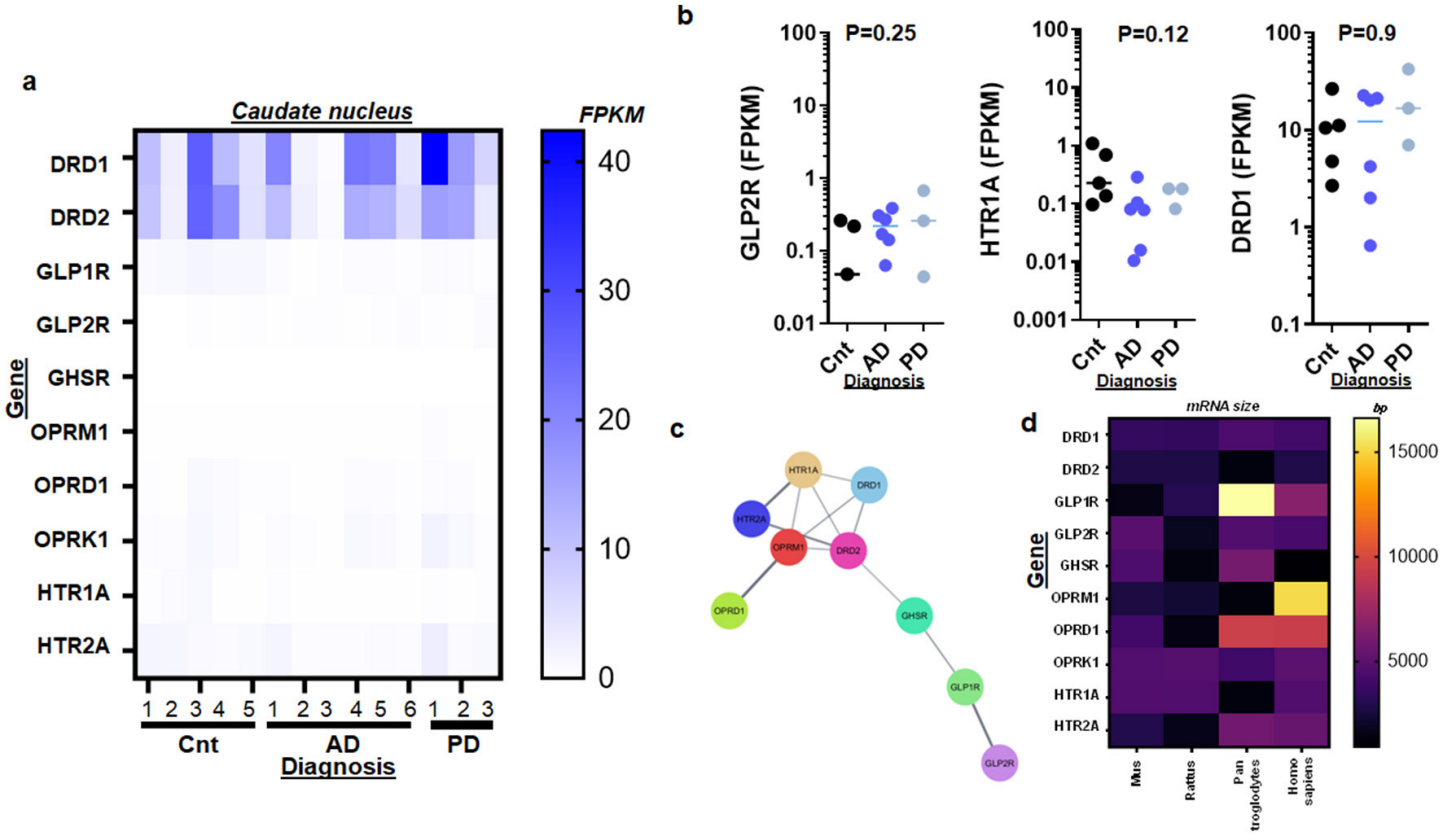
Neurotransmitter receptor genes which dopamine, GLP-1, opioid, and serotonin can activate in the aged caudate nucleus with AD and/or PD. **(a)** Heat map illustrating an overall view of whole transcriptome RNA-Seq, involving DRD1, DRD2, and eight other genes in control (CNT, *n* = 5), Alzheimer's disease (AD, *n* = 6), and Parkinson's disease (PD, *n* = 3). **(b)** Scatter plots showing RNA activities of GLP-2R, HTR1A, and DRD1 in the caudate nucleus with AD and PD as compared to that of control (Cnt) specimens. Statistical analysis by Mann-Whitney test. **(c)** Protein network of genes shown in **(a, b)**. Note that a flag represents statistical significance. **(d)** Heat map summarizing an overall view of mRNA sizes of ten genes shown in a represented by genes directly and indirectly linked to DRD1 over mouse (Mus), rat (Rattus), chimpanzee (Pan troglodytes), and human (Homo sapiens) chromosome. Note that on average RNA/transcript size is at least twice as long (up to 15,000 bp) than those in Supplementary Figure S11b (up to 8,000 bp).

**Figure 6:**
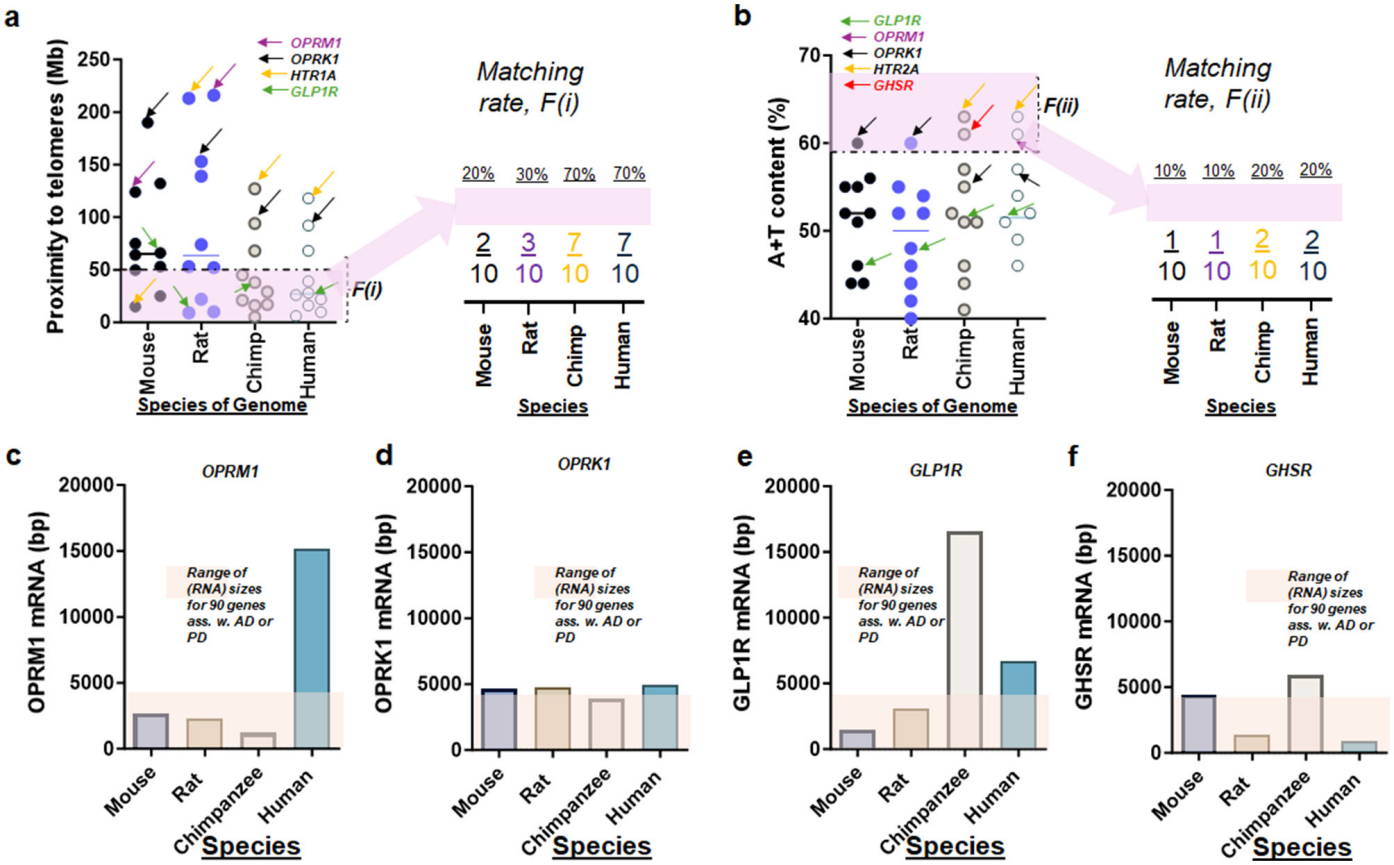
Relative mutability of genes encoding neurotransmitters in four species. **(a)** Left-Proximity to telomeres, F(i), of ten genes encoding neurotransmitter receptors over four species of mouse, rat, chimpanzee, and human chromosome. Right-summary of the matching rate between genes of interest and F(i). The dashed dotted line indicating the threshold for proximity to telomeres at 50 Mb. **(b)** Left-A+T content, F(ii), of ten genes encoding neurotransmitter receptors over four species of mouse, rat, chimpanzee, and human chromosome. Right-summary of the matching rate between genes of interest and F(ii). The dashed dotted line indicating the threshold for high A+T content at 59% [the average A+T content of human chromosomes according to Nusbaum et al. (2006)]. **(c)** Bar graph summarizing base-pair (bp) length of OPRM1 transcript (RNA), suggesting unusually long size in humans over four species. **(d)** Bar graph summarizing sizes of OPRK1 transcript (RNA), suggesting consistent sizes over four species. **(e)** Bar graph summarizing sizes of GLP-1R transcript (RNA), suggesting unusually long size in chimpanzees over four species. **(f)** Bar graph summarizing sizes of GHSR transcript (RNA), suggesting moderately varying sizes over four species. Rectangles in color covering bars of each plot indicating range of (RNA) sizes for 90 genes associated with (ass. w.) AD or PD **(c–f)**.

Contrary to expectations, only GLP-1R showed significant alterations in RNA activities of AD and/or PD samples compared to controls (Figures 2a, 5a–d). This suggests that among the neurotransmitters studied, the pathways mediated by GLP-1 may play a pronounced role in the pathophysiology of these diseases. Moreover, an intriguing aspect of our findings was the unusual length of nucleotide sequences observed in certain transcripts. Specifically, GLP-1R transcripts (Figure 6e) were notably longer in chimpanzees, and OPRM1 transcripts were elongated in humans compared to those in rodent chromosomes (Figure 6c). This variation in transcript size across species could indicate evolutionary differences in gene regulation and expression, potentially influencing neurotransmitter function and susceptibility to neurodegenerative conditions (Figure 6).

In the genomic landscape of neurotransmitter receptor genes linked to neurodegeneration in disorders such as AD and PD, distinct patterns of genetic stability emerge. Notably, the opioid receptor gene (OPRK1) and the serotonin receptor gene (HTR1A) demonstrated genomic characteristics suggesting a lower likelihood of harboring deleterious mutations. Specifically, OPRK1 and HTR1A in chromosomes of chimpanzees and humans did not exhibit proximity to telomeres [Factor *F*(i)] or high adenine and thymine (A+T) content [Factor *F*(ii)] (Figures 6a, b). For example, OPRK1 genes are located distant (>50 Mb) from their telomeres in all four species (OPRK1), while GLP-1R genes demonstrate genetic instability by proximity to telomeres when the base pair size of GLP-1R in mice (>50 Mb) is compared to that in rats, chimps, and humans (all three <50 Mb; Figure 6a). Species-dependent difference of GLP-1R genes is further substantiated by their transcript sizes as well (Figure 6e; varying over species) when compared with OPRK1 (Figure 6d; constant over species).

In contrast, GLP-1R gene displayed a higher susceptibility to mutations, meeting the proximity to telomeres criterion [*F*(i)] in rat, chimpanzee, and human chromosomes (Figure 6a). This suggests potential for higher mutation rates in GLP-1R, which could influence its evolutionary adaptability and function. Furthermore, other neurotransmitter genes including DRD1, DRD2, GLP2R, GHSR, OPRD1, and HTR2A showed moderate mutability, particularly in chimpanzees and humans. These genes had a 70% matching rate to mutation-associated factors, compared to only 20 or 30% in mice and rats (Figure 6a, right panel), highlighting species-specific differences in genetic stability. However, these genes rarely satisfy the high A+T content criterion, with poor matching rates of 10 or 20% (Figure 6b, right panel). Interestingly, despite the variability in nucleotide size among neurotransmitter receptor genes, OPRM1 exhibited an unusual nucleotide size in humans similar to that observed in GLP-1R, underscoring potential vulnerabilities in these receptors (Figure 6d). However, genes encoding eight other neurotransmitter receptors, such as OPRK1 and GHSR, demonstrated consistent RNA sizes across the four species of interest, suggesting a degree of genetic conservation (Figure 6d).

## Discussion

In a subcortical or relatively deep zone compared to subarachnoid space (Shim and Madsen, 2018; Shim et al., 2019), one would wonder if there is clear sign of neuroinflammation or protection against the inflamed neurons in the aged human brain. Our study demonstrated that NFkb1 (P50 or class I NF-κB family) was elevated in the CN of AD and that of PD patients (Figure 2g). NF-κB transcription factors are crucial for CNS processes like neurogenesis, neuritogenesis, and synaptic plasticity related to learning and memory (Levenson et al., 2004; Shih et al., 2015). NF-κB activation protects neurons from excitotoxicity, oxidative stress, and amyloid β toxicity, while overexpression of p65 (class II NF-κB family) rescues apoptotic neurons. In astroglia and microglia, NF-κB regulates brain injury responses, inflammation, and blood–brain barrier function, contributing to neurodegenerative disorders (Shih et al., 2015).

It has been published recently that clinical trials using GLP-1R agonists were promising in treating AD patients with cognitive decline and brain shrinkage through liraglutide, and motor symptoms of PD patients through exendin-4 (Byetta, Bydureon), liraglutide (Victoza, Saxenda), and lixisenatide (Lyxumia, Adlyxin) (Athauda et al., 2017; Foltynie and Athauda, 2020; Holscher, 2022, 2024). However, the molecular mechanisms underlying the improvements in cognition and motor function observed in Phase II trials remain to be determined. The results presented in this study provide insights into how GLP-1R might influence mitochondrial dynamics in AD and PD.

The CN—an area crucial for motor and cognitive functions—exhibits significant molecular alterations (Figures 1–4) that contribute to the pathogenesis of these neurodegenerative disorders. Understanding how these changes are linked is the key to the molecular mechanisms driving AD and PD (Kinney et al., 2018; McGregor and Nelson, 2019). Decreasing GLP-1R availability (Barrett et al., 2024) in the CN impacts cellular signaling (Rowlands et al., 2018), mitochondrial function (Tomas et al., 2011; Morales et al., 2014), and endosomal trafficking (Fang et al., 2020). GLP-1R plays a key role in neuroprotection, metabolism, and cellular homeostasis (Nadkarni et al., 2014; Reich and Holscher, 2022). Reduced GLP-1R signaling impairs mitochondrial function (Boland et al., 2020) by decreasing SLC25A6 (a mitochondrial ADP/ATP transporter) expression (Palmieri, 2013; Ding et al., 2023), leading to compromised ATP production and overall dysfunction (Clemencon et al., 2013).

This mitochondrial impairment disrupts communication with the cell nucleus (Kotiadis et al., 2014; Saki and Prakash, 2017), which typically regulates stress responses and metabolism through mitochondrial signals like ATP and reactive oxygen species (Dawson et al., 1993; Babizhayev, 2011; Banh et al., 2016; Resende et al., 2022). Such disruption affects nuclear gene expression (Cheng et al., 2011; Calarco et al., 2021; Yang et al., 2022), further impairing mitochondrial health and cellular stress adaptation (Venkataraman et al., 2022). The deployment of PCA on extensive RNA-seq data from human postmortem tissues has highlighted significant mitochondrial involvement (Figure 1). The identification of genes such as FKBP5 and MT-ATP6P1 as highly variable along the PC1 not only emphasizes the role of mitochondria in neurodegeneration but also suggests potential targets for therapeutic intervention. The mitochondrial dysfunction implied by MT-ATP6P1 is central to the pathological landscape of both AD and PD, driving processes that may exacerbate disease progression (Monzio Compagnoni et al., 2020). In the rat model of AD (Faborode et al., 2022), FKBP5 was elevated consistently with our findings (Figures 1, 4b). FKBP5 was best-known for stress response (Levy-Gigi et al., 2013; Mahon et al., 2013; Buchmann et al., 2014; Kitraki et al., 2015; Kohrt et al., 2015; Bishop et al., 2018, 2021; Cox et al., 2021; Hausl et al., 2021; Richter et al., 2021; Asadi-Pooya, 2023; Everson et al., 2023) but more recently suggested as stress driver (Maiaru et al., 2023). However, when it comes to oxidative stress, which can connect FKBP5 to AD and PD alike, there has been few study concerning environmental toxin-induced oxidative stress in the lung cancer cell line (Meng et al., 2022). More study on FKBP5 is warranted related to oxidative stress in human specimens of AD and those of PD.

Additionally, SLC9A9 (NHE9), an endosomal Na+/H+ exchanger, is upregulated (Figure 2c), which may represent a compensatory response to impaired GLP-1R signaling and mitochondrial dysfunction (Donowitz et al., 2013). Elevated SLC9A9 can lead to altered endosomal pH, impacting vesicular trafficking, cargo sorting, and receptor recycling (Pedersen, 2024). This imbalance in endosomal function exacerbates cellular stress and homeostasis issues (Wong et al., 2019). The interconnected effects of reduced GLP-1R RNA (Figure 2f) culminate in insufficient SLC25A6 expression (Figure 2g), impaired mitochondrial-nuclear communication (Ryan and Hoogenraad, 2007), and elevated SLC9A9 expression (Figure 2h), contributing to cellular stress, energy imbalance, and dysregulated trafficking (Schirrmacher, 2020), ultimately affecting neuronal health and function (Devine and Kittler, 2018; Murali Mahadevan et al., 2021; Trigo et al., 2022).

Because TNF-related inflammatory pathway was ranked #1 or 2 in AD and PD alike (Figures 1c–e; Supplementary Figures S1, S2), we sought RNA markers representing inflammation in these disorders. In addition to NFkB1 (Figure 2g), we found that NFE2L2, belonging to the TNF pathway (ranked top 4 in Figure 1e), was significantly elevated in the CN of AD and that of PD, respectively, as compared to controls (Figure 2f). It has been suggested that stress-dependent activation of NFE2L1 occurs primarily through post-translational regulation, specifically via the KEAP1-mediated pathway (Hellyer et al., 2021; Arolt et al., 2023; Hijazo-Pechero et al., 2023; Paik et al., 2023; Wu et al., 2023). The gene set analysis (Figures 1c–e) showing a strong link between the TNF pathway and NFE2L2 (NRF2) expression in AD and PD is supported by previous reports (Pajares et al., 2016; Eldesoqui et al., 2023; Ghany et al., 2023; Janahmadi et al., 2023), emphasizing inflammation and oxidative stress (Mohamed et al., 2022). The TNF pathway's high ranking in both conditions (Figure 1e; Supplementary Figures S1e, S2e) suggests heightened inflammation in the brain, with TNF-α driving chronic inflammation that can worsen neuronal damage and contribute to disease progression. Elevated NFE2L2 RNA levels in the CN indicate a compensatory response to this oxidative stress. NFE2L2 plays a key role in activating antioxidant genes (Pajares et al., 2016), suggesting that the cells are attempting to mitigate damage. However, despite this increased NFE2L2 encoding NRF2 activity, inflammation and oxidative stress may still overwhelm the protective mechanisms, contributing to further neuronal injury.

Moreover, the analysis of RNA activities of additional risk genes such as SORL1 (Maple-Grodem et al., 2018) and PLCG2 (van der Lee et al., 2019, 2020) further enriches our understanding of AD and PD. The elevated RNA levels of SORL1 and PLCG2 in disease conditions point toward their active participation in the disease's molecular framework, possibly through pathways involved in amyloid processing (Reitz et al., 2011) and immune responses (Claes et al., 2022), respectively. In dissecting the broader implications of stress-related gene alterations, the significant increases in RNA activities of genes like FKBP5 reinforce the notion of a stress driving mechanism (Maiaru et al., 2023) in AD and PD (Yap et al., 2013; Lemche, 2018). Such findings highlight the potential vulnerability of the aging brain to external and internal stressors, which could accelerate the pathogenesis of neurodegenerative diseases. In a combined statistical analysis of AD/PD, Trem2 demonstrated significant differences, partly because of the similar range of each sample group (AD: 10-40; PD: 12-39 FPKM). When separated as AD (*n* = 6) and PD (*n* = 3), respectively (Figure 3b lower panel), however, no significance was detected in Trem2 due to limited sample size.

The exploration of neurotransmitter dynamics through the analysis of receptors such as DRD1, DRD2, and others, and the specific alterations observed in GLP-1R and HTR1A underscore the complexity of neurotransmitter interactions in neurodegeneration. The unique transcript size findings further emphasize the role of genetic factors in mediating neurotransmitter effects, which could influence disease outcomes.

Overall, detecting statistically significant marker genes in different human specimens was challenging. In Supplementary material alone, only 12 of 52 genes ranked initially in the top 100 of session 1 (Supplementary Figures S4–S15) demonstrated statistical significance when combined with session 2. Among these (12 of 52), six specific markers (Supplementary Figures S12e, f, S13a) were just reconfirmations of the prior report related to hemoglobin genes and related signaling molecules. The observation of reduced GLP-1R RNA activities in the CN aligns with findings of disrupted mitochondrial and endosomal functions (Monzio Compagnoni et al., 2020), reflected in the downregulation of key solute carrier proteins such as SLC25A6 and SLC37A1. This disruption hints at a broader impact of diminished GLP-1 signaling on cellular energy homeostasis and waste processing (Guglielmi and Sbraccia, 2017), critical areas affected in neurodegenerative diseases. The concurrent increasing trend of the heat shock protein gene HSPA2 could be interpreted as an adaptive response to increased proteostatic stress, a common feature in the degenerating brain (Leak, 2014). This study's small sample size reflects limited availability of well-characterized postmortem brain tissue. While results are biologically meaningful, they remain exploratory. Ongoing work includes expanding the cohort and validating key genes via qPCR and protein assays, with future large-scale analyses.

In conclusion, this study provides transcriptomic evidence linking mitochondrial dysfunction, inflammation, and altered stress signaling in the caudate nucleus of individuals with AD and PD. Elevated expression of NF-κB1 and NFE2L2 (NRF2), alongside reduced levels of SLC25A6 and GLP-1R, points to a compensatory but insufficient response to oxidative stress and impaired mitochondrial function. The observed decrease in HBA1 (hemoglobin subunit alpha) expression further suggests compromised oxygen transport, which may exacerbate neuronal vulnerability. Additionally, increased PLCG2 expression implicates microglial activation and immune signaling in disease progression. Our findings reinforce the central role of TNF-α signaling and mitochondrial dysfunction in AD and PD pathology and support the potential therapeutic relevance of GLP-1R agonists, particularly in restoring energy homeostasis and reducing neuroinflammation. While several candidate biomarkers—such as FKBP5, SORL1, and SLC9A9—were identified, the small sample size limits definitive conclusions. Nonetheless, this work lays a foundation for further validation studies and highlights the caudate nucleus as a key region for mechanistic insights and therapeutic targeting in neurodegeneration.

## Methods

### Human postmortem tissues collection

Postmortem tissues were sourced from the National Institute of Health (NIH) NeuroBioBank (NBB), USA, over a 2-year period. The tissues consisted primarily of caudate nucleus samples obtained from aged individuals, preserved in a frozen state through various NIH NBB repositories (White et al., 2022; Barrett et al., 2024). These samples were subsequently transported to our laboratory for analysis. According to the NBB records, the caudate nucleus (DeVito et al., 2007; Deshpande et al., 2009; Jang et al., 2017; Peterson et al., 2019) specimens were collected within a postmortem interval averaging 16 ± 8 h, ranging from 4 to 25 h after death. The study included samples from 5 unaffected controls, 6 individuals diagnosed with Alzheimer's disease (AD), and 3 with Parkinson's Disease (PD), as detailed in the inclusion criteria and diagnostic categories (Supplementary Tables S1, S2). The cohort comprised four male and ten female specimens, with sex demographics specified in Supplementary Table S2.

### Bulk RNA-seq

Two separate sessions of whole transcriptome RNA-Seq were conducted, analyzing a total of 62,704 gene loci across a sample size of *N* = 14 (*n* = 5 for control and *n* = 6 for AD; *n* = 3 for PD). From the total data set, 3.4% (2,144 out of 62,704 loci) were statistically significant at *p* < 0.05. The sorting criteria were based on *p*-value and effect size, with genes encoding hemoglobin subunit proteins emerging as the most significant (*p* = 0.000000000101). This systematic approach in RNA-Seq data analysis enables a focused examination of gene variations potentially pivotal in understanding disease mechanisms.

### Total RNA isolation

Total RNA was isolated from the caudate nucleus of three different groups: unaffected controls, CH cases in the elderly, and AD cases, utilizing the QIAsol-based RNA isolation kit (RNeasy Lipid Tissue Mini Kit, QIAGEN) according to the prior reports (Shim et al., 2013, 2016, 2019; White et al., 2022; Barrett et al., 2024). Briefly, tissue samples (50 mg) were homogenized in QIAzol Lysis reagent. Chloroform is added, and the mixture is centrifuged to separate it into aqueous and organic phases. The upper aqueous layer is collected, and ethanol is added to optimize binding conditions. The sample is transferred to an RNeasy spin column, in which up to 100 μg of total RNA bind to the membrane while phenol and other contaminants were washed away. Finally, high-quality RNA was eluted using 40 μl of RNase-free water. The quality and concentration of the isolated RNA were measured using a NanoDrop spectrophotometer (Thermofisher).

### Principal component analysis (PCA)

Raw data from the Bulk RNA-Seq was organized in a data sheet of GraphPad Prism (version 10.2.3) software: control and disease (PD) sample were arranged at column 1 through 6, for example if one compared three controls and three disease samples, in which the response was FPKM readings shown on raw data of the Bulk RNA-seq. Then, we analyzed data using Principal Component Analysis (PCA) under “Multiple Variable Analyses”. We selected the columns of interest to analyze such as column A, B, C, … J, K, L (Select PCs on Eigen values option). Once the PCA plot such as Loadings and PC scores was obtained, we saved the image in the temporary memory (buffer) and pasted in the Power point file to generate plots.

We performed PCA on combined AD and PD datasets vs. controls to identify shared transcriptomic alterations and potential pan-neurodegenerative biomarkers (Figure 1a). Despite distinct pathologies, AD and PD share mechanisms like neuroinflammation, oxidative stress, hypoxia, and synaptic dysfunction. By analyzing AD, PD, and combined groups separately, we aimed to compare disease-specific and overlapping molecular patterns. The combined PCA helped reveal consistent gene expression changes across both conditions, supporting the identification of shared pathways and candidate biomarkers. This analysis complements, rather than replaces, individual disease comparisons and adds an integrative layer for understanding common neurodegenerative mechanisms.

### Gene set enrichment and hierarchical clustering analysis

Gene set enrichment analysis (GSEA) was conducted using GSEA 4.3.3 and G-profiler to identify significant pathways and gene sets. For organizing and interpreting the RNA-Seq dataset, hierarchical clustering was performed, generating dendrograms via the Instant Clue software. These methods facilitated a structured analysis of gene expression patterns, enhancing the understanding of biological functions and interactions within the dataset.

### Calculation of genomic proximity to telomeres and nucleotide compositions

To assess the proximity of specific genes to telomeres and calculate the percentage of adenine and thymine (A + T) content in nucleotides, we utilized the NCBI Genome Data Viewer (https://www.ncbi.nlm.nih.gov/genome/gdv/) for humans, chimpanzees, rats, and mice and the publicly available GC Content Calculator (https://www.biologicscorp.com/tools/GCContent/#.XvctCi-z2uV). These tools facilitated accurate measurement of A + T content as a percentage and provided comprehensive details on the total base-pair lengths of nucleotides. This approach supports precise genomic characterization, aligning with findings from recent studies (Lucas et al., 2021; McKnight et al., 2021; Raines et al., 2022; White et al., 2022; Hart et al., 2023; McKnight et al., 2023; Barrett et al., 2024).

### Assessment of two factors associated with high mutation rates

The relationship between gene location and mutation rates in human chromosomes has been extensively documented, previously (Nusbaum et al., 2006), in which the biological underpinnings of heightened mutation rates near telomeres were described. Following their methodology, we have adopted a factor associated with high mutation rates to determine the proximity of genes to their respective telomeres. This study has mapped the location of seven genes across mouse, rat, chimpanzee, and human chromosomes to estimate each gene's adenine and thymine (A + T) content and their telomeric proximity. These efforts are part of our broader aim to better understand genomic vulnerabilities linked to telomeric regions (Raines et al., 2022; Hart et al., 2023; Barrett et al., 2024). The theoretical basis of this estimation is based on the following assumptions:

(i) If the recombination frequency is 50 centimorgans (cM) or less, the genes are considered linked.
(ii) If the recombination frequency exceeds 50 cM, the genes are considered unlinked.

Additionally, it is noted that 1 cM approximately equals 1 million bases (Mb), as established previously (Hastbacka et al., 1992).

## Statistical analysis

We utilized statistical methods and visualization tools from Prism (GraphPad Software Inc.) to analyze the data. This software facilitated the creation of heatmap plots and bar graphs, using data obtained from the genome data viewer and GC content calculator. Due to the nature of our data, we opted for non-parametric tests, which are more conservative compared to parametric tests that assume random treatment assignment and a Gaussian distribution. Specifically, we used independent *t*-test for comparisons between two groups and multiple comparisons after Brown-Forsythe and Welch ANOVA test for comparisons among three groups with respect to the control, unless noted otherwise. Differences were considered statistically significant at *P* < 0.05. *P*-values are detailed in the figures and legends, denoted as ^*^P < 0.05, ^**^P < 0.01, and ^***^P < 0.005.

## Data Availability

The datasets presented in this study can be found in online repositories. The names of the repository/repositories and accession number(s) can be found in the article/Supplementary material.
