# Supplementary material for: NFκB1: a common biomarker linking Alzheimer's and Parkinson's disease pathology
Source: Front Neurosci. 2025 May 6;19:1589857. doi: 10.3389/fnins.2025.1589857 (PMC12089106; doi:10.3389/fnins.2025.1589857)
Supplement: Supplementary file 1 [file Data_Sheet_1.pdf]

Supplementary Material for

NFκB1: A Common Biomarker Linking Alzheimer's and Parkinson's Disease Pathology

Adam Cunningham[1], Emma Barrett[1], Sebastian Risch[1], Peter H.U. Lee[2,3], Chan Lee[4], Abhay Moghekar[5], Prabir Patra[1], Joon W. Shim[1]*

Correspondence to: shim@marshall.edu

This file includes:

Figure S1 to S17;
Tables S1 to S3

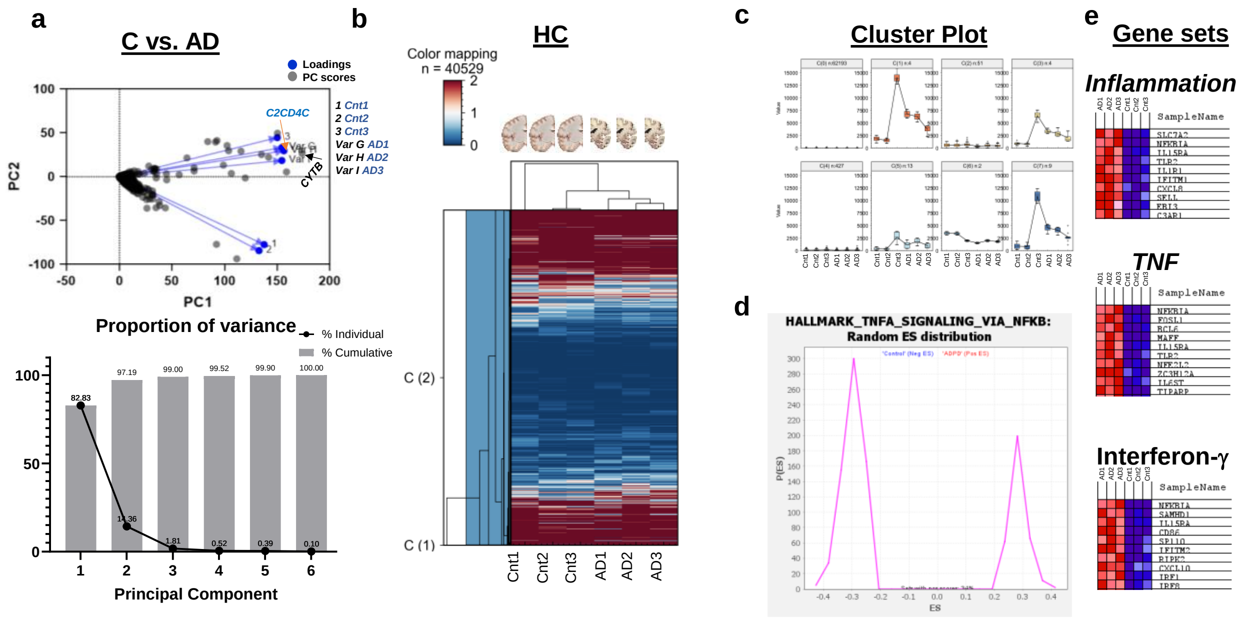

Figure S1. Integrated Analysis of Gene Expression Profiles in Alzheimer's Disease: PCA, Hierarchical Clustering, and Gene Set Enrichment Analysis

(a) top: PCA biplot of caudate nucleus specimens from control and subjects with AD. Regarding PC scores: A datapoint with a high positive score on PC1 (CYTB) indicates that it aligns strongly with the pattern or trend represented by PC1. Regarding Loadings: If the original variables X1 and X2 have high positive loadings on PC1, a datapoint (i.e. C2CD4C) that is far right on PC1 is likely to have high values for X1 and X2. Bottom: Proportion of variance plot showing PC1 accounting for 82.8 % of variances. (b) Analysis of 40,529 data points from gene expression profiles of caudate nucleus specimens revealed two primary clusters, C(1) and C(2), displaying distinct gene expression patterns between three control and three AD specimens. (c) Gene expressions across eight subgroups (C(0) through C(7)) was analyzed for both control and AD specimens. The plots illustrate differential expression trends within each subgroup, helping to identify specific molecular mechanisms potentially contributing to AD pathology. (d) Distribution of enrichment scores for the hallmark inflammatory response was analyzed, showing variability and frequency of genes associated with inflammation across samples. This analysis underscores the role of inflammatory signaling pathways in AD. (e) Expression levels of key gene sets related to Inflammation, TNF, and Interferon-Gamma were compared between control and AD specimens. Differential expression patterns were observed, with AD specimens showing heightened expression in these pathways, highlighting their potential role in AD's pathological features. This comprehensive gene expression analysis emphasizes the critical genetic and molecular distinctions between control and AD specimens, particularly in relation to mitochondrial function, inflammatory response, and overall cellular mechanisms.

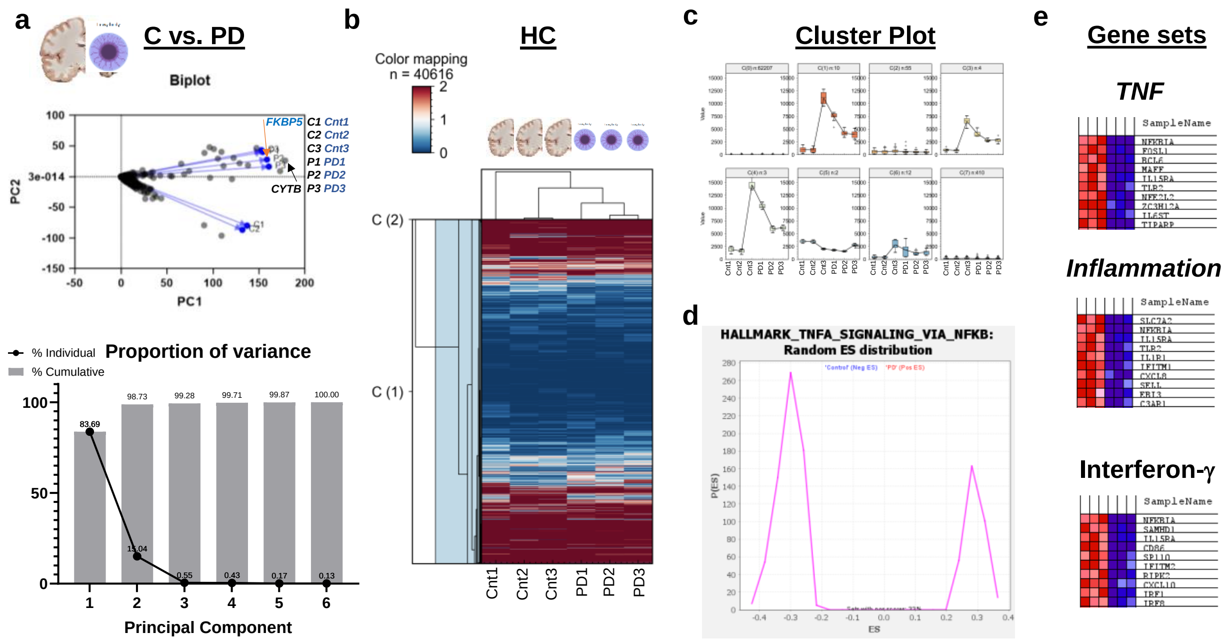

Figure S2. Integrated Analysis of Gene Expression Profiles in Parkinson's Disease: PCA, Hierarchical Clustering, and Gene Set Enrichment Analysis

(a) top: PCA biplot of caudate nucleus specimens from control and subjects with PD. Regarding PC scores: A datapoint with a high positive score on PC1 (CYTB) indicates that it aligns strongly with the pattern or trend represented by PC1. Regarding Loadings: If the original variables X1 and X2 have high positive loadings on PC1, a datapoint (i.e. FKBP5) that is far right on PC1 is likely to have high values for X1 and X2. Bottom: Proportion of variance plot showing PC1 accounting for 83.7 % of variances. (b) Hierarchical Clustering of Gene Expression Profiles from Control and PD Specimens: This figure presents a hierarchical clustering dendrogram based on the analysis of 40,616 data points from gene expression profiles of caudate nucleus specimens. The x-axis denotes the samples involved in the study. This analysis aids in visualizing the genetic distinctions between control and AD groups, potentially highlighting key pathways involved in the disease's pathogenesis. (c) Cluster Plot Analysis of Gene Expression Across Eight Subgroups in Control and PD Specimens (d) Distribution of Enrichment Scores (ES) for Hallmark TNF alpha signaling (e) Comparative Analysis of Gene Expression in Inflammation, TNF, and Interferon-Gamma Pathways Between Control and Alzheimer's Disease (AD) Specimens: This figure illustrates the expression levels of three key gene sets—TNF, Inflammation, and Interferon-Gamma—in control and PD specimens.

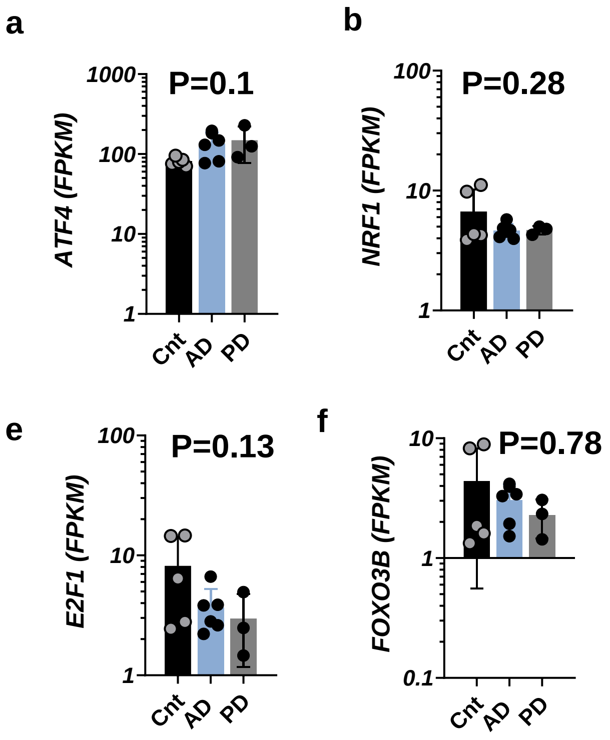

Figure S3. Transcription factors reported to mediate GLP-1 signaling, neuroinflammation, and mitochondria dysfunction (see also Fig. 2) (a) Scatter plots with bar graphs showing ATF4 RNA activity in the caudate nucleus of AD and that of PD as compared to controls. (b) Scatter plots with bar graphs showing NRF1 RNA activity in the caudate nucleus of AD and that of PD as compared to controls. (c) Scatter plots with bar graphs showing CREB1 RNA activity in the caudate nucleus of AD and that of PD as compared to controls. (d) Scatter plots with bar graphs showing PPARGC1A RNA activity in the caudate nucleus of AD and that of PD as compared to controls. (e) Scatter plots with bar graphs showing E2F1 RNA activity in the caudate nucleus of AD and that of PD as compared to controls. (f) Scatter plots with bar graphs showing FOXO3B RNA activity in the caudate nucleus of AD and that of PD as compared to controls. (g) Scatter plots with bar graphs showing Hif1alpha RNA activity in the caudate nucleus of AD and that of PD as compared to controls. (h) Scatter plots with bar graphs showing SP1 RNA activity in the caudate nucleus of AD and that of PD as compared to controls.

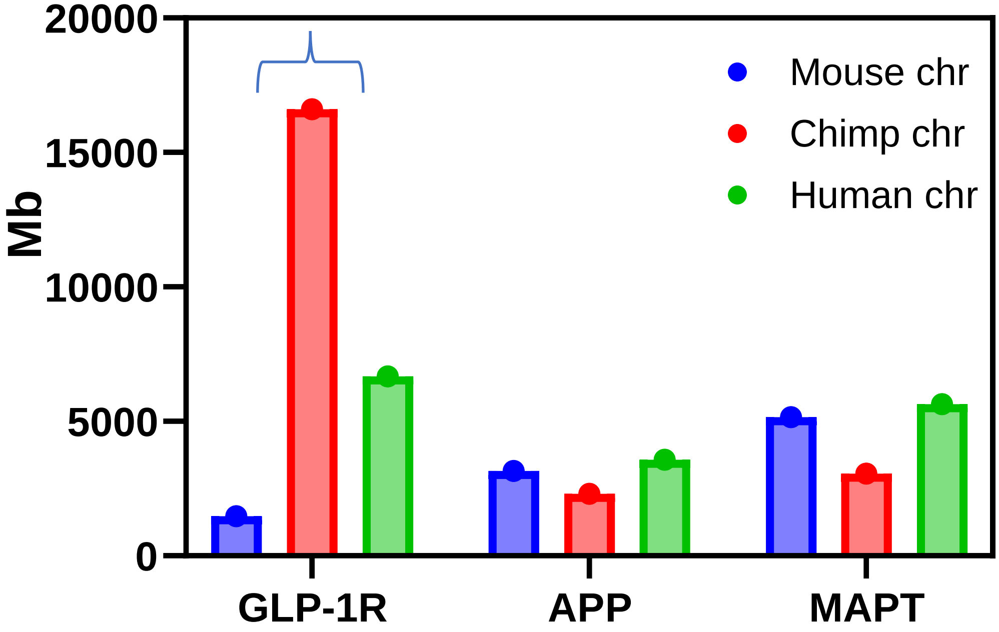

Figure S4. Nucleotide size of GLP-1R, APP, and MAPT transcript in three species Bar graphs showing nucleotide size in million bases (Mb) of GLP-1R, APP, and MAPT in mouse, chimpanzee (chimp) and human chromosome (chr). Bracket indicating an unusually long GLP-1R RNA size in chimp

GSEA: C v PD

Enrichment in phenotype: PD (3 samples)
•38 / 50 gene sets are upregulated in phenotype **PD**
•36 gene sets are significant at FDR < 25%
•29 gene sets are significantly enriched at nominal pvalue < 1%
•31 gene sets are significantly enriched at nominal pvalue < 5%
•Snapshot of enrichment results
•Detailed enrichment results in html format
•Detailed enrichment results in TSV format (tab delimited text)
•Guide to interpret results

Enrichment in phenotype: Control (3 samples)
•12 / 50 gene sets are upregulated in phenotype **Control**
•8 gene sets are significantly enriched at FDR < 25%
•4 gene sets are significantly enriched at nominal pvalue < 1%
•6 gene sets are significantly enriched at nominal pvalue < 5%
•Snapshot of enrichment results
•Detailed enrichment results in html format
•Detailed enrichment results in TSV format (tab delimited text)
•Guide to interpret results

GSEA: C v AD

Enrichment in phenotype: AD (3 samples)
•39 / 50 gene sets are upregulated in phenotype **AD**
•37 gene sets are significant at FDR < 25%
•29 gene sets are significantly enriched at nominal pvalue < 1%
•32 gene sets are significantly enriched at nominal pvalue < 5%
•Snapshot of enrichment results
•Detailed enrichment results in html format
•Detailed enrichment results in TSV format (tab delimited text)
•Guide to interpret results

Enrichment in phenotype: Control (3 samples)
•11 / 50 gene sets are upregulated in phenotype **Control**
•7 gene sets are significantly enriched at FDR < 25%
•4 gene sets are significantly enriched at nominal pvalue < 1%
•6 gene sets are significantly enriched at nominal pvalue < 5%
•Snapshot of enrichment results
•Detailed enrichment results in html format
•Detailed enrichment results in TSV format (tab delimited text)
•Guide to interpret results

GSEA: C v ADPD

Enrichment in phenotype: ADPD (6 samples)
•38 / 50 gene sets are upregulated in phenotype **ADPD**
•36 gene sets are significant at FDR < 25%
•32 gene sets are significantly enriched at nominal pvalue < 1%
•33 gene sets are significantly enriched at nominal pvalue < 5%
•Snapshot of enrichment results
•Detailed enrichment results in html format
•Detailed enrichment results in TSV format (tab delimited text)
•Guide to interpret results

Enrichment in phenotype: Control (3 samples)
•12 / 50 gene sets are upregulated in phenotype **Control**
•8 gene sets are significantly enriched at FDR < 25%
•5 gene sets are significantly enriched at nominal pvalue < 1%
•6 gene sets are significantly enriched at nominal pvalue < 5%
•Snapshot of enrichment results
•Detailed enrichment results in html format
•Detailed enrichment results in TSV format (tab delimited text)
•Guide to interpret results

Figure S5. GSEA* Results Comparing Control vs. Disease States. The analysis reveals significant gene set upregulation and enrichment in PD (38/50 sets upregulated), AD (39/50 sets upregulated), and AD+PD (38/50 sets upregulated) samples. For PD, 36 sets are significant at FDR < 25%, with 29 and 31 sets enriched at p-values < 1% and < 5% respectively. In AD, 37 sets meet the FDR < 25% threshold, with 29 and 32 sets enriched at p-values < 1% and < 5%. ADPD shows 36 sets with FDR < 25%, and 32 and 33 sets enriched at p-values < 1% and < 5%. Enrichment analyses highlight key pathways involved in disease mechanisms, available in detailed HTML and TSV formats.

** This GSEA text summary succinctly encapsulates the enrichment results, detailing the significant gene sets and their levels of enrichment across different disease states, facilitating quick interpretation of the genetic underpinnings observed in these neurodegenerative conditions.*

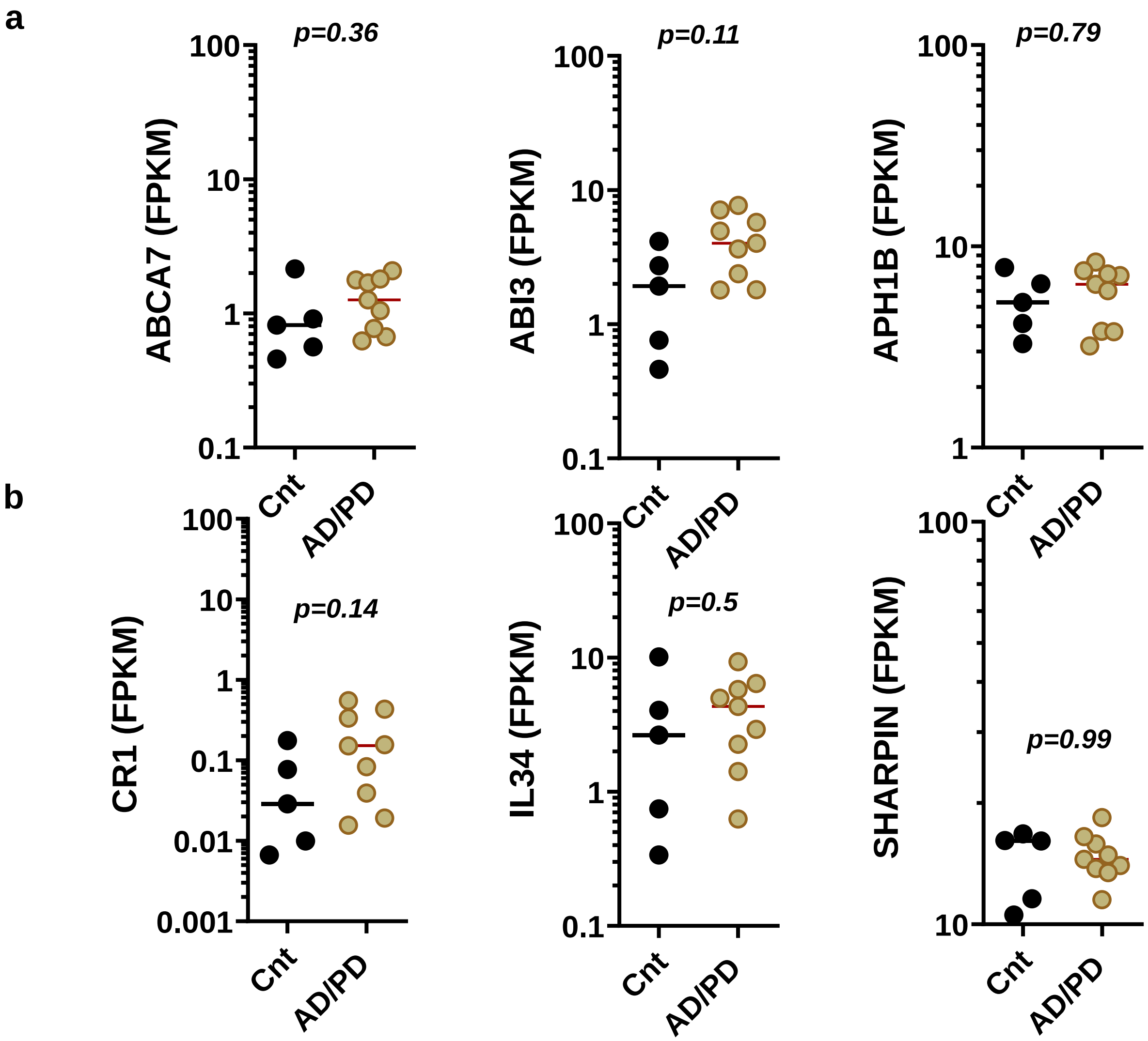

Figure S6. Genes previously reported to form a genetic landscape of AD (a-b) Risk variants or alleles included in the polygenic risk score in the prior study associated with AD (Scheltens et al., 2021) compared in the AD-PD pooled samples with control (Cnt) group. Note that none of these genes reported previously in Fig. 3 of the said report (Scheltens et al., 2021) demonstrate statistical significances between the groups.

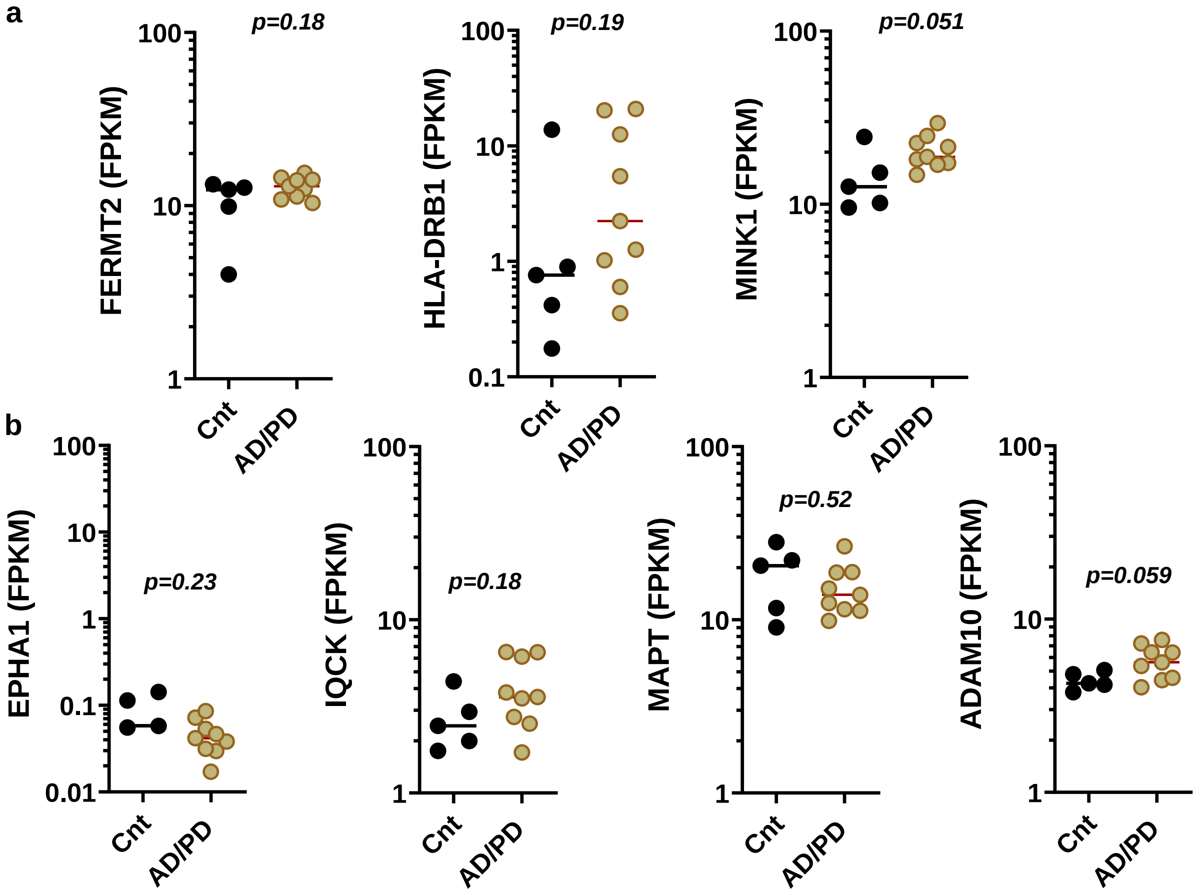

Figure S7. RNA activities of candidates previously reported as risk genes associated with AD (a-b) Risk variants or alleles included in the polygenic risk score in the prior study associated with AD (Scheltens et al., 2021) compared in the AD-PD pooled samples with control (Cnt) group.

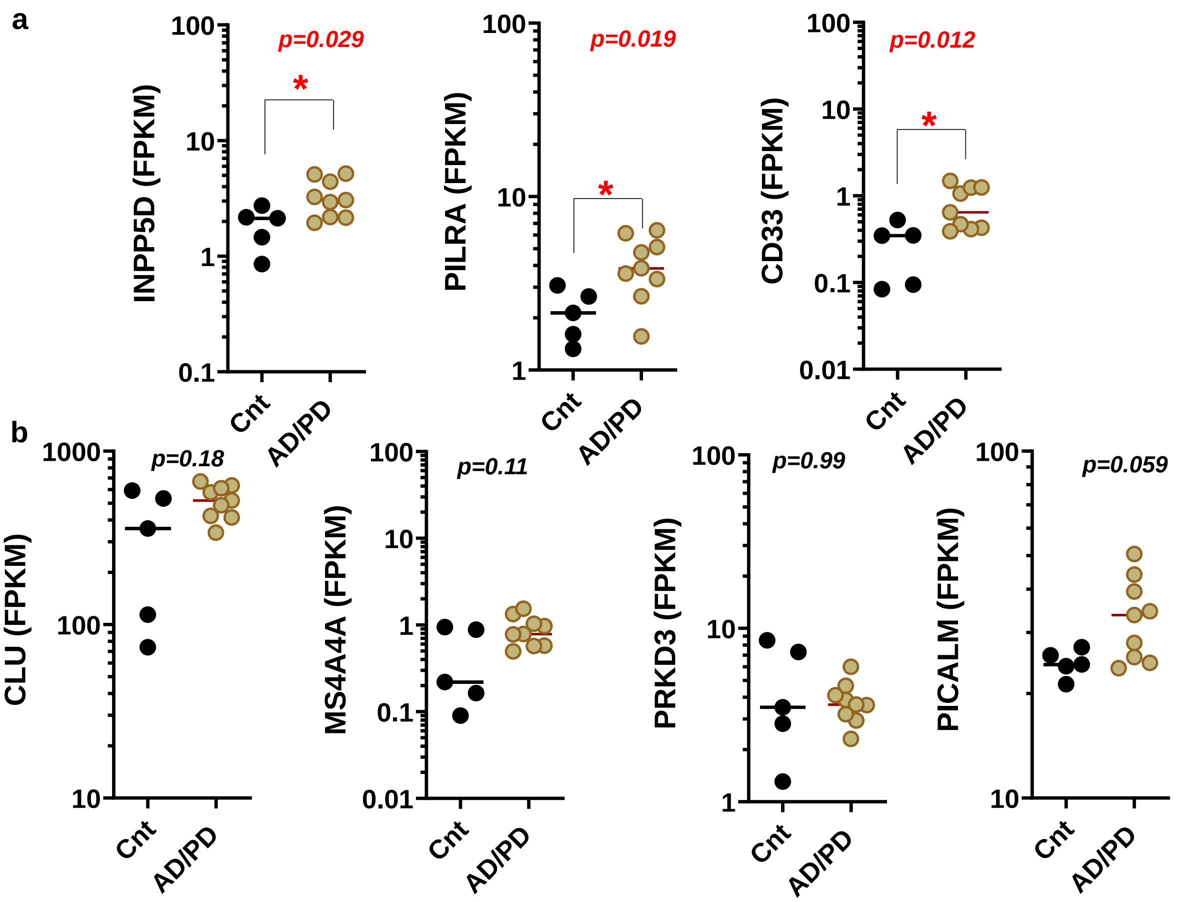

Figure S8. RNA activities of risk genes previously reported to be associated with AD (a-b) Risk variants or alleles included in the polygenic risk score in the prior study associated with AD (Scheltens et al., 2021) compared in the AD-PD pooled samples with control (Cnt) group.

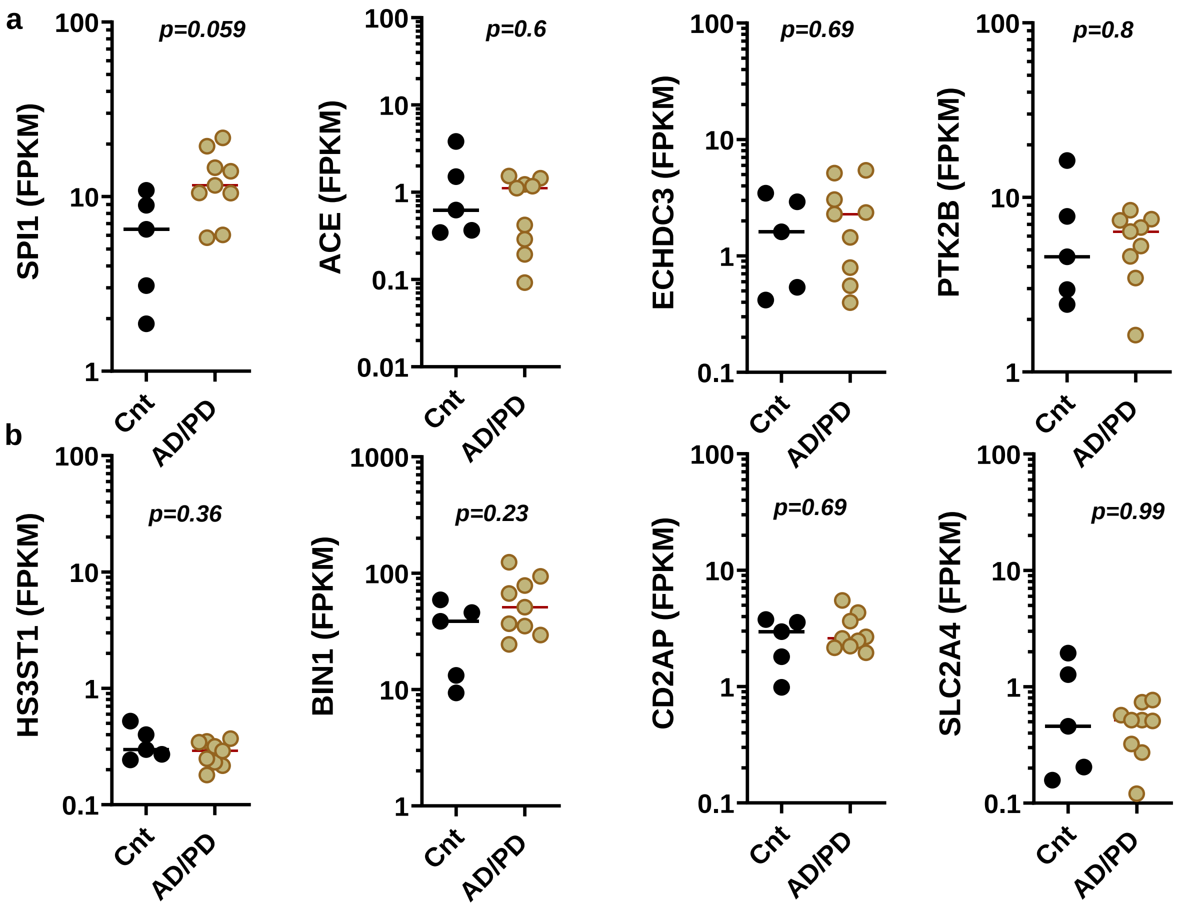

Figure S9. RNA activities of risk genes previously reported to be associated with AD (a-b) Risk variants or alleles included in the polygenic risk score in the prior study associated with AD (Scheltens et al., 2021) compared in the AD-PD pooled samples with control (Cnt) group.

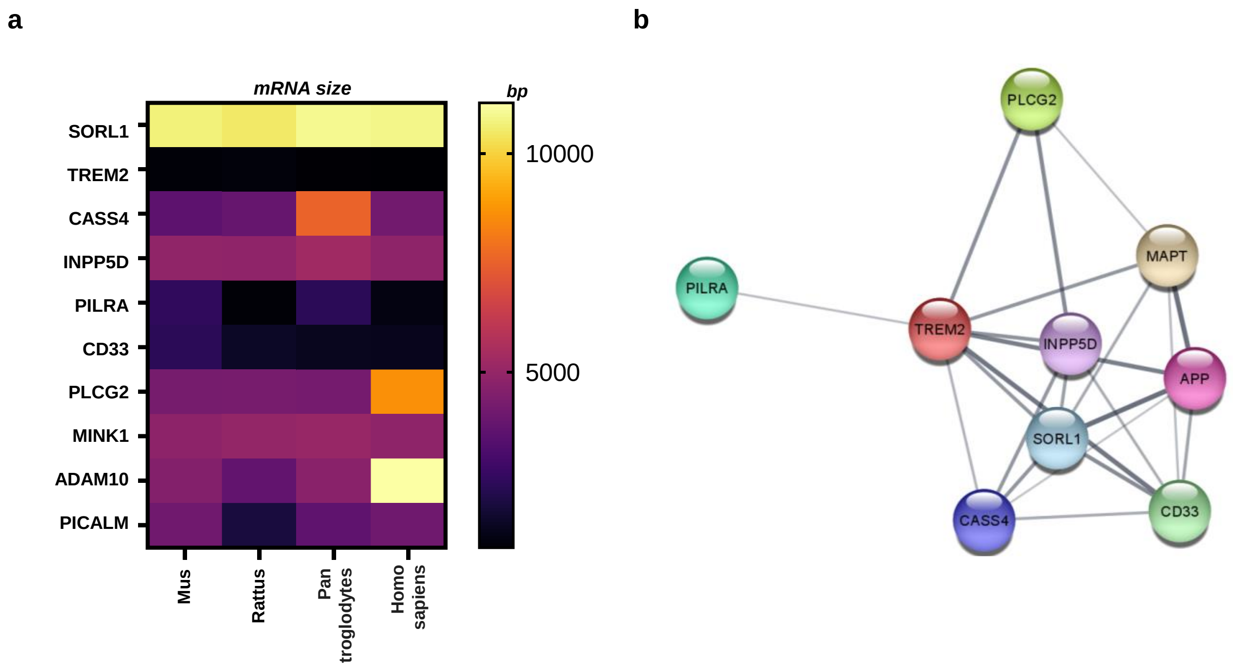

Figure S10. Transcript sizez of AD risk genes and protein network associated with AD (a) Heat map illustrating transcript sizes of ten genes shown in Fig. 3 represented by the previously known risk genes of AD over mouse (Mus), rat (Rattus), chimpanzee (Pan troglodytes), and human (Homo sapiens) chromosome. (b) SORL1-TREM2 driven protein network

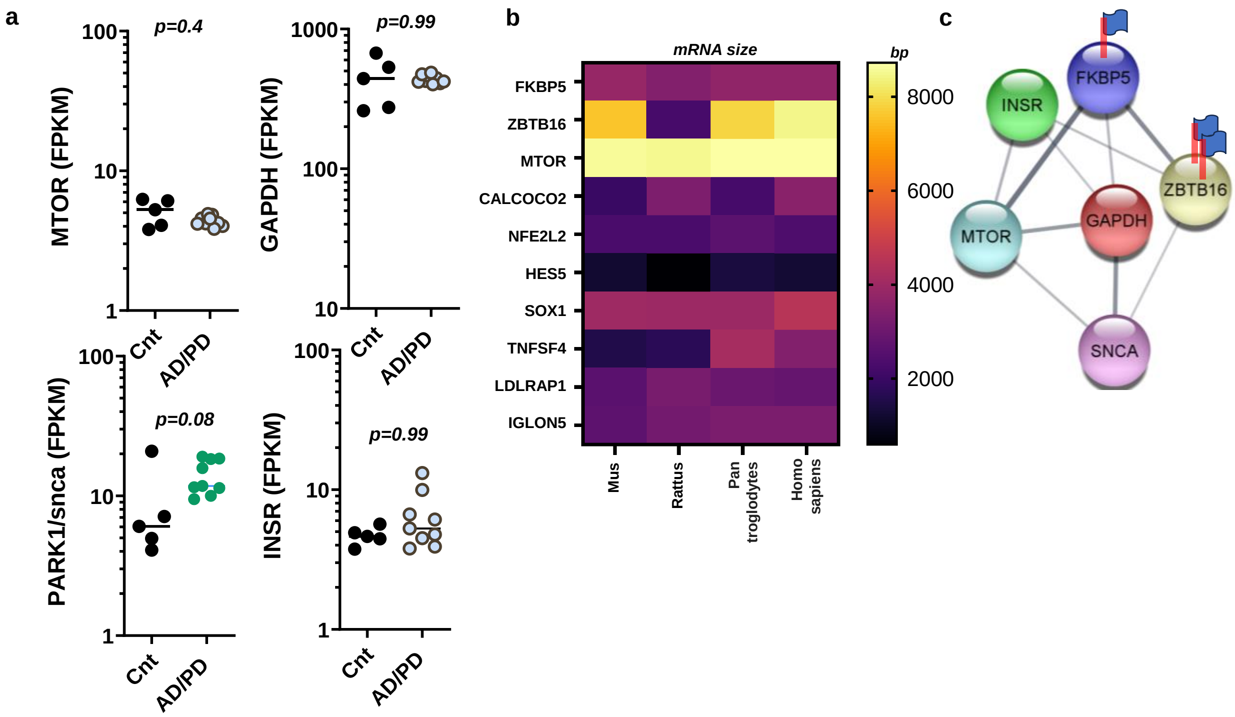

Figure S11. MTOR-associated genes in the aged brain with AD/PD (a) the scatter plots summarizing confirmation of the previous report (elevated FKBP5 in AD) and the manifestation of neuropsychiatric symptoms through elevated ZBTB16 in the caudate nucleus (striatum) of the aged brain with AD/PD as compared to those of control (Cnt) obtained from the whole transcriptome RNA-Seq (see also Fig.4a-c). (b) Heat map showing transcript (mRNA) sizes of ten genes shown in Fig. 4 over mouse (Mus), rat (Rattus), chimpanzee (Pan troglodytes), and human (Homo sapiens) chromosome (c) a network chart showing associations between FKBP5, ZBTB16, and genes linked to mTOR involving INSR, GAPDH, and SNCA (see also Fig.4a-c). Note that two mRNAs showing significant alterations in AD/PD were highlighted with flags, consistent with scatter plots in a.

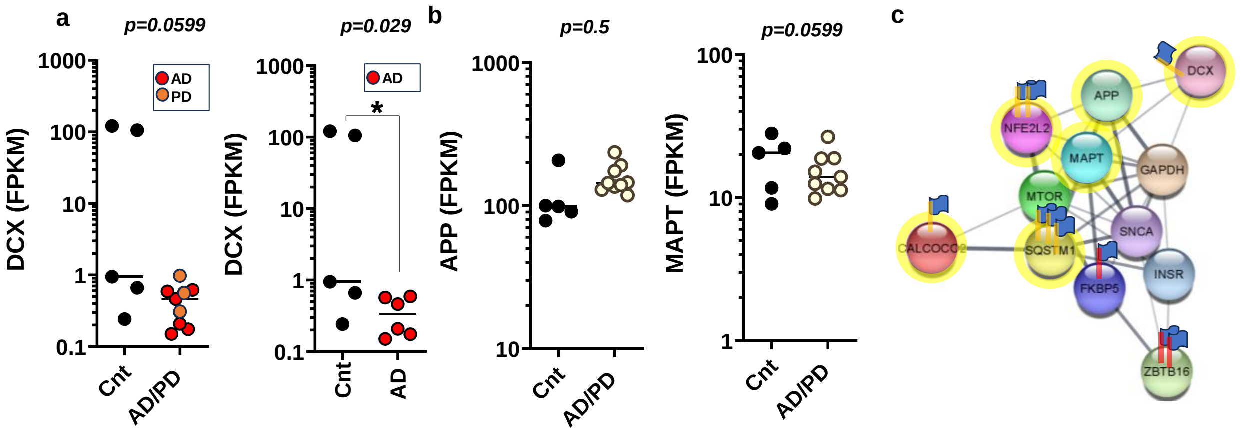

Figure S12. RNA activities of DCX, APP, and MAPT in the aged brain with AD/PD (a) the scatter plots exhibiting DCX in the caudate nucleus of the aged brain with AD/PD (left) as compared to those of Cnt. While the pooled sample failed to show a significant difference between the groups, there was a significant reduction in AD alone (right) as reported previously (Barrett et al., 2024) (b) the scatter plots showing APP (p=0.5) and MAPT mRNA (p=0.0599) in the caudate nucleus of the aged brain with AD/PD (a-c); *, $p<0.05$; **, $p<0.01$; ***, $p<0.005$ (a-c) (c) a network chart showing associations between SQSTM1 and five other genes (highlighted in circular yellow) along with prior genes shown in Fig. S10. Note that mRNAs showing significant alterations in AD/PD were highlighted with flags in upright positions. The flag in DCX is tilted as the statistical significance is only found in AD compared to controls (b and d).

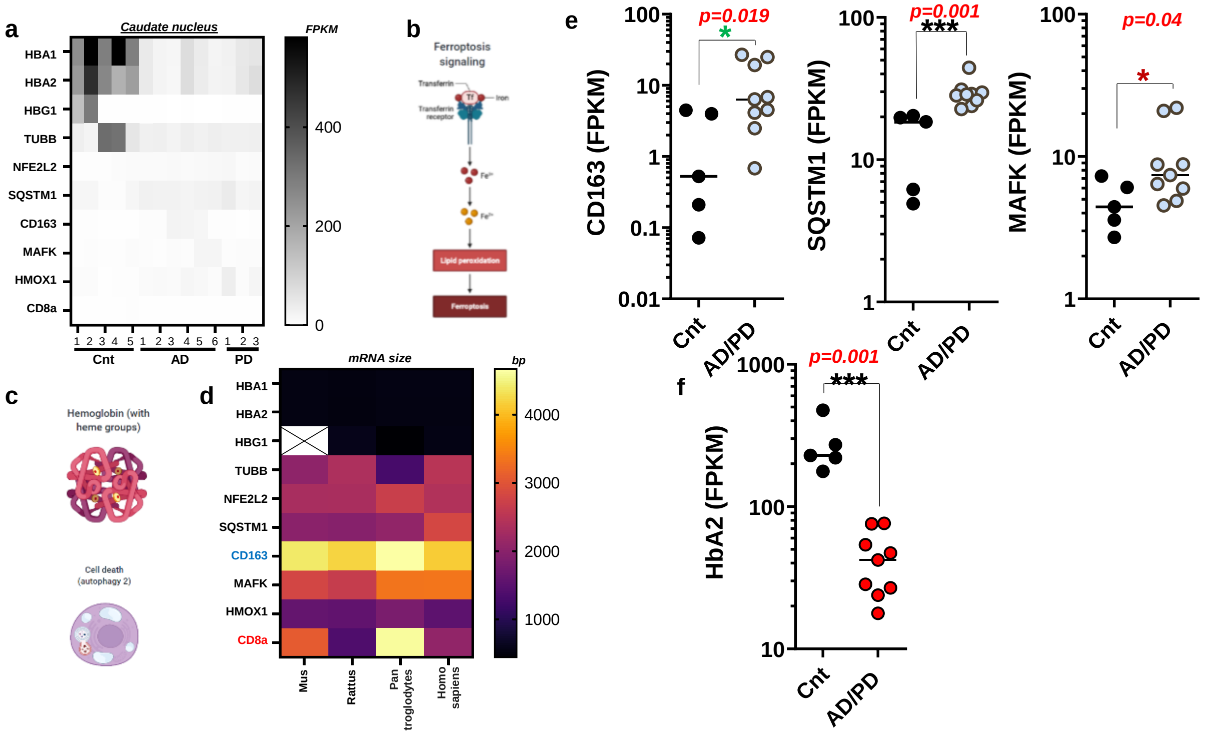

Figure. S13 Cerebral RNA activities suggesting responses to oxidative stress, escalated autophagy, and reduced hemoglobin in the aged caudate nucleus with AD and/or PD (a) the heat map summarizing an overall view of whole transcriptome RNA-Seq, involving HBA1 and nine other genes in the caudate nucleus of control (CNT, n=5), Alzheimer's disease (AD, n=6), and Parkinson's disease (PD, n=3). (b) A diagram showing the recently discovered cell death mechanism (Dixon et al., 2012) through the iron-dependent accumulation of lipid peroxides to lethal levels or ferroptosis. (c) Simplified illustrations showing hemoglobin with heme groups (top) and another cell death mechanism or autophagy (bottom) (d) the heat map illustrating mRNA sizes of ten genes shown in the left (a) represented by HBA1 and others over mouse (Mus), rat (Rattus), chimpanzee (Pan troglodytes), and human (Homo sapiens) chromosome. (f) Scatter plots showing RNA activity of HBA2 in the caudate nucleus with AD/PD as compared to that of control (Cnt) specimens.

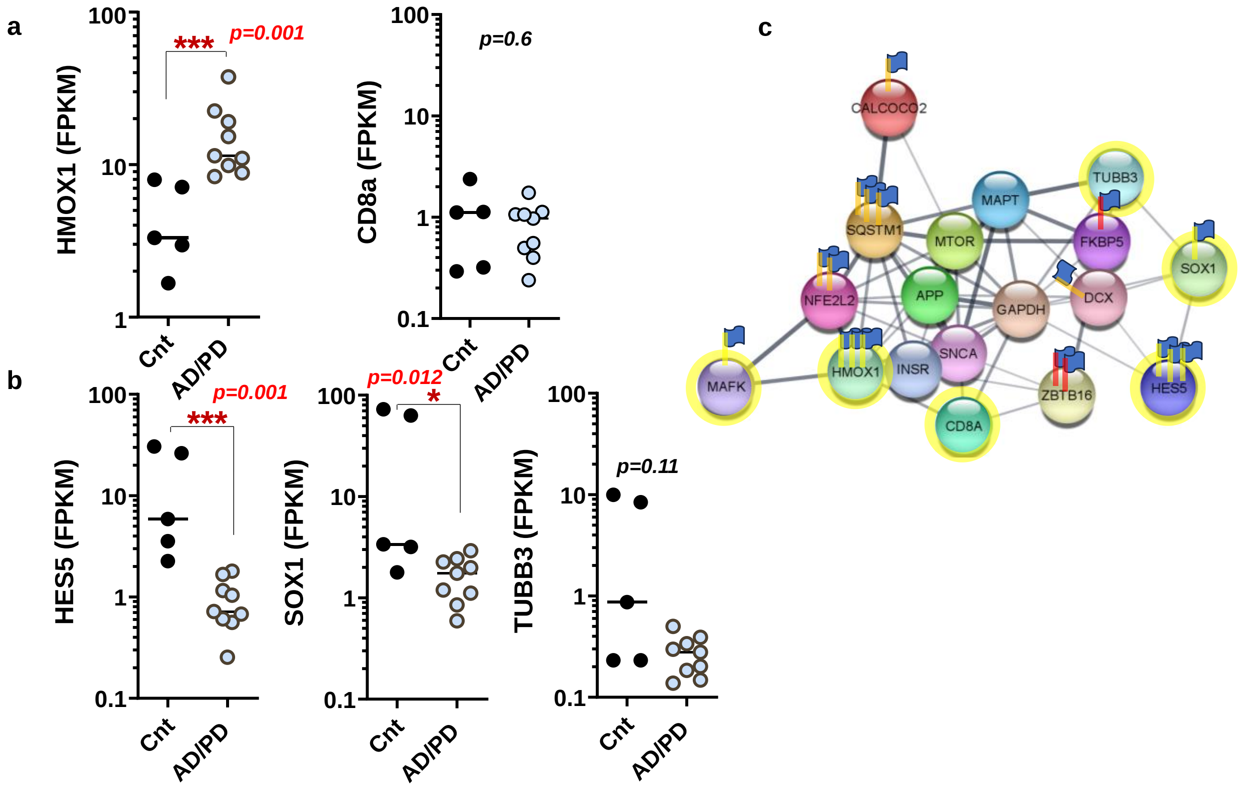

Figure. S14 Elevated anti-oxidant (HMOX1) and oxidative stress (MAFK) along with markers for reduced neuronal cell differentiation (HES5 and SOX1) in the aged brain with AD/PD (a) the scatter plots showing elevated mRNA expressions of HMOX1 (p=0.001) and MAFK (p=0.04) in the caudate nucleus of the aged brain with AD/PD as compared to those of Cnt obtained from the whole transcriptome RNA-Seq. (b) the scatter plots summarizing reduced mRNA expressions of HES5 (p=0.001) and SOX1 (p=0.012) in the caudate nucleus with AD/PD as compared to those of Cnt. Note that Cd8a (p=0.6) and Tubb3 (p=0.11) did not differ between the groups (a-b). (c) a network chart showing associations between HMOX1 and five other genes (highlighted in circular yellow) along with prior genes shown in Fig. 2-3. Note that mRNAs showing significant alterations in AD/PD were highlighted with upright flags, in which number of flags are consistent with statistical significances shown in a-b. *, p<0.05; **, p<0.01; ***, p<0.005.

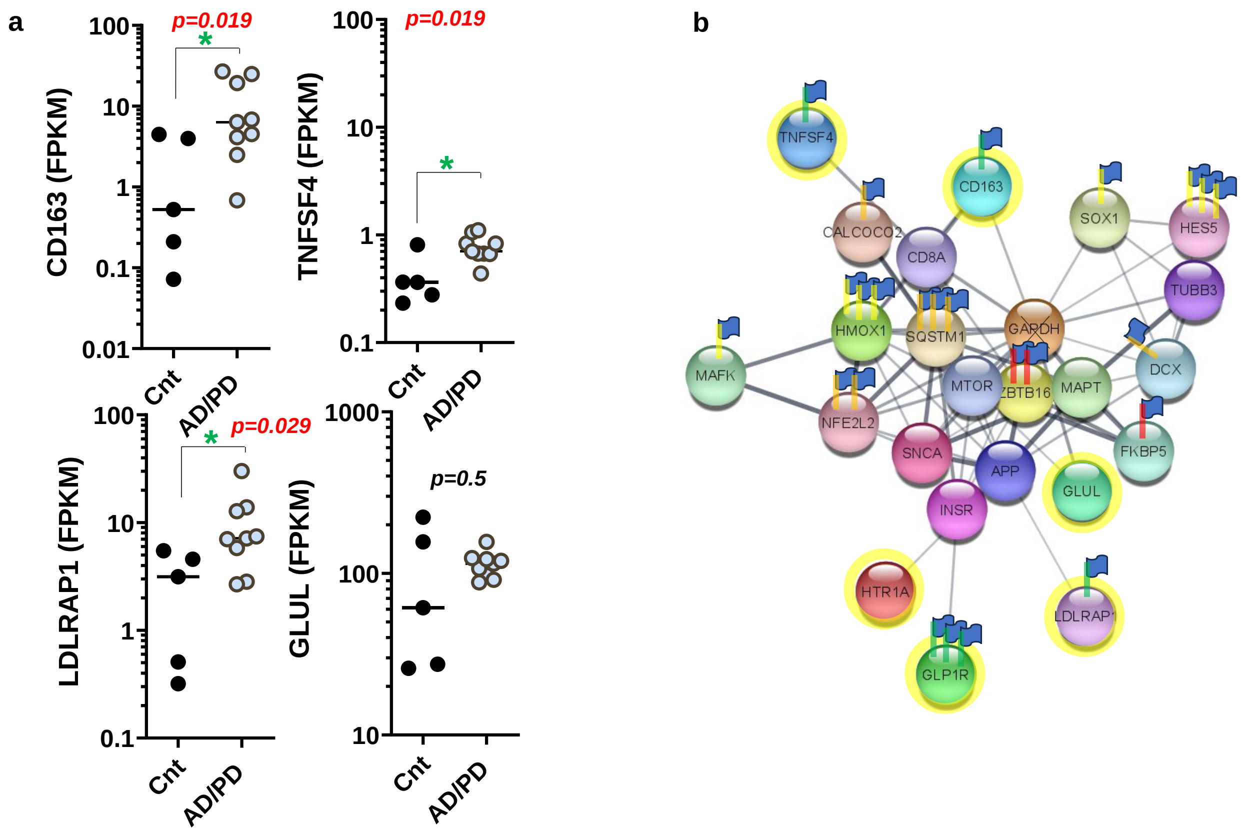

Figure. S15 Altered mRNAs associated with glucose dysmetabolism, inflammation, and dyslipidemia in the aged brain with AD/PD (a) the scatter plots showing differential regulations of GLP1R (p=0.001), CD163 (p=0.019), TNFSF4 (p=0.019), and LDLRAP1 mRNA (p=0.029) in the caudate nucleus of the aged brain with AD/PD as compared to those of Cnt obtained from the whole transcriptome RNA-Seq. Note that GLUL (p=0.5) and HTR1A mRNA (p=0.059) did not differ between the groups. (b) a network chart summarizing associations between GLP1R and five other genes (highlighted in circular yellow) along with prior genes shown in Fig. 2-4. Note that mRNAs showing significant alterations in AD/PD were highlighted with flags, consistent with statistical significances shown in a. *, p<0.05; **, p<0.01; ***, p<0.005 by Mann-Whitney test.

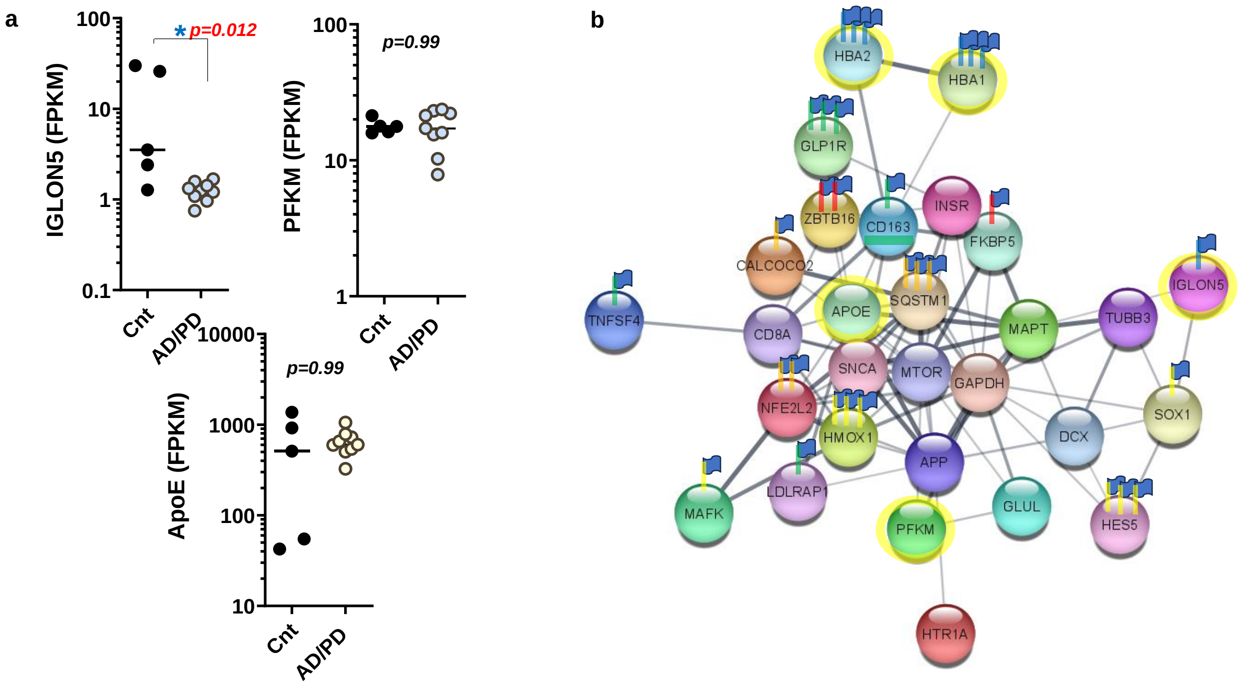

Figure. S16 Impaired IGLON5 and hemoglobin genes in the aged brain with AD/PD (a) the scatter plots showing decreases of IGLON5 (p=0.012), HBA1 (p=0.0001), and HBA2 (p=0.001) in the caudate nucleus of the aged brain with AD/PD as compared to those of Cnt obtained from the whole transcriptome RNA-Seq. Note that PFKM (p=0.9) and APOE mRNA (p=0.9) did not differ between the groups. (b) a network chart summarizing associations between IGLON5 and four other genes (highlighted in circular yellow) along with prior genes shown in Fig. 2-5. Note that mRNAs showing significant alterations in AD/PD were highlighted with flags, consistent with statistical significances shown in a. *, p<0.05; **, p<0.01; ***, p<0.005 by Mann-Whitney test.

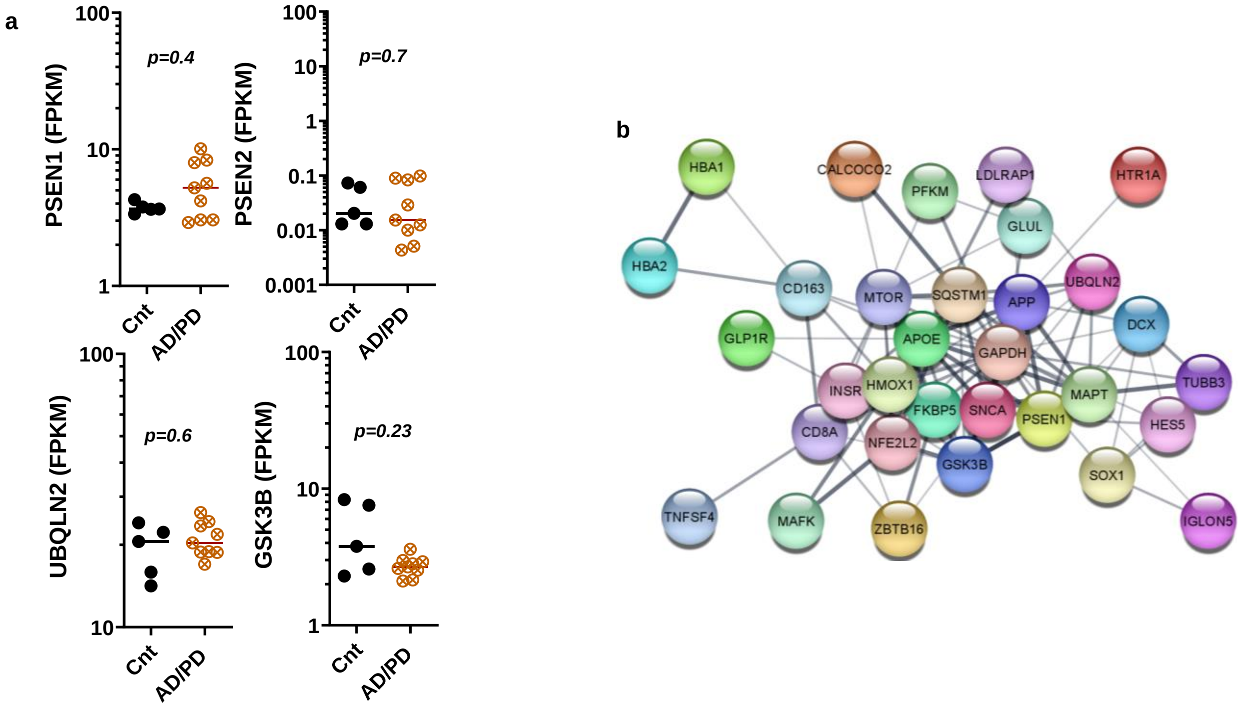

Figure s17. Select AD risk genes in the aged brain with AD/PD (a) the scatter plots showing RNA activities of PSEN1, PSEN2, UBQLN2, and GSK3B in the caudate nucleus with AD/PD as compared to that of controls. Note that these four genes did not differ between the groups. (b) a network chart summarizing associations between FKBP5, and other genes shown in Fig. S5-S16.

Table S1 Inclusion criteria: human postmortem tissues from the Neurobiobank

Criteria	Tissue	RNA integrity number (RIN)	Age	Type	Sex	HIV*	HBSAG**	PMInterval***
Include	Cortex, caudate nucleus, cerebellum	7 – 10	≥ 65 yr	Frozen	Male & female	Negative	Negative	< 36 hr
Exclude	elsewhere	<7	< 65 yr	Fixed	-	Positive	Positive	≥ 36 hr

* Human immunodeficiency virus; ** Hepatitis B Surface Antigen Test; *** Postmortem interval

Table S2 Postmortem specimen information

Numbering	Subject ID	Age (years)	Disorder	Sex	Race	Medical History
1	5219	76	Unaffected Control	Female	White	
2	4921	73	Unaffected Control	Female	White	* gall bladder problem
3	4789	72	Unaffected Control	Female	White	
4	5671	78	Unaffected Control	Male	White	
5	s06424	71	Unaffected Control	Female	White	
6	1212	72	AD	Female	White	
7	5584	88	AD	Female	White	
8	5501	78	AD	Female	White	
9	6560	67	AD	Female	White	
10	5914	83	AD	Male	White	
11	5683	69	AD	Female	White	
12	1311	72	PD	Male	White	
13	1272	78	PD	Male	White	
14	1401	80	PD	Female	White	

* Donor also with high blood pressure. She collapsed while in the hospital. She was still breathing when found on the floor but with no spontaneous respiration and no cardiac pulse. No further medical history available. "

Table S3 Primer sequences for *human* gene transcripts

**PDK4** (product size: 249 b); Exon 2<br>(upstream) TCAGCCTTCCCTTACACCAA<br>(downstream) ACAAGTCATCAAAGCCACACA	**GLP-1R** (product size: 118 b); Exon 7<br>(upstream) CTCATGCAGTACTGTGTGGC<br>(downstream) TCACGTAGAGCCTGAAGATCC
**LDLRAP1** (product size: 213 b); Exon 2<br>(upstream) TGTTTGCATACATCGCCCAG<br>(downstream) GGTTCCCAGAGATTTCACCC	**GAPDH** (product size: 201 b); Exon 6<br>(upstream) ACCCAGAAGACTGTGGATGG<br>(downstream) TTCTAGACGGCAGGTCAGGT

References for supplementary material

Barrett, E., Ivey, G., Cunningham, A., Coffman, G., Pemberton, T., Lee, C., et al. (2024). Reduced GLP-1R availability in the caudate nucleus with Alzheimer's disease. *Front Aging Neurosci* 16**,** 1350239. doi: 10.3389/fnagi.2024.1350239.

Dixon, S.J., Lemberg, K.M., Lamprecht, M.R., Skouta, R., Zaitsev, E.M., Gleason, C.E., et al. (2012). Ferroptosis: an iron-dependent form of nonapoptotic cell death. *Cell* 149(5)**,** 1060-1072. doi: 10.1016/j.cell.2012.03.042.

Scheltens, P., De Strooper, B., Kivipelto, M., Holstege, H., Chetelat, G., Teunissen, C.E., et al. (2021). Alzheimer's disease. *Lancet* 397(10284)**,** 1577-1590. doi: 10.1016/S0140-6736(20)32205-4.
